# Influence of Choline-Based Ionogel on Transdermal Delivery of Vancomycin Hydrochloride

**DOI:** 10.1021/acs.molpharmaceut.5c00255

**Published:** 2025-05-16

**Authors:** Deepanjan Datta, Sony Priyanka Bandi, Venkata Vamsi Krishna Venuganti

**Affiliations:** † Department of Pharmaceutics, Manipal College of Pharmaceutical Sciences, 76793 Manipal Academy of Higher Education, Manipal 576104 Karnataka State, India; ‡ Department of Pharmacy, Birla Institute of Technology and Science (BITS) Pilani, Hyderabad Campus, Hyderabad 500078 Telangana State, India

**Keywords:** choline-geranate, oleic acid, palmitic acid, pluronic F-127, ionogel, vancomycin hydrochloride, dermal delivery

## Abstract

Ionic liquids (ILs) have attracted considerable interest as new drug delivery solvents because of their superior transdermal absorption of large molecular weight drugs across the biological barrier and their capacity to solubilize hydrophobic compounds. It is difficult to administer hydrophilic peptide treatments with large molecular weights through the skin. Vancomycin hydrochloride (VH) is a glycopeptide antibiotic that cures bacterial infections. With a molecular weight of 1449 Da and a high water solubility of 50 mg/mL, VH is an impressive compound. This study aimed to quantify the amount of VH transdermal penetration by analyzing the influence of choline-based ILs. Choline geranate was used as an IL in this investigation, whereas oleic acid (OA) (unsaturated) and palmitic acid (PA) (saturated) were chosen as the two types of fatty acids. The molar ratio of choline bicarbonate (CB) to either OA (CO) or PA (CP) was 2:1. In an additional series of trials, choline geranate (CAGE) ILs were prepared by mixing CB and geranic acid in a 1:2 molar ratio. For NMR spectroscopy, powder X-ray diffraction, differential scanning calorimetry, and Fourier transform infrared investigations, these formulations were characterized. Zeta potential indicated that all of the formulations had negative charges. The decreased irritation potential of CAGE, as shown by conductivity studies, makes it appropriate for skin application. To determine which formulation enhancers worked best, ex vivo skin permeation studies were carried out on both intact and tape-stripped skin. After 48 h, skin transport investigations showed that neat VH did not penetrate the excised porcine skin. Nevertheless, the CO and CP-based formulations greatly improved the skin penetration (6729 ± 437 μg/cm^2^) and retention (3892 ± 215 μg/g) of VH across the tape-stripped skin, whereas CAGE exhibited the most significant improvement (*p* < 0.05). Ionogel (CAGE-P) was finally fabricated by combining CAGE with 22.7% w/v Pluronic F-127 and 45.0% w/v PEG-400. The physical and rheological properties of VH-loaded CAGE-P gel were examined. The amount of VH permeated across the CAGE-P gel cotreated intact skin was 369 ± 41 μg/cm^2^, but that penetrated tape-stripped skin was 7543 ± 585 μg/cm^2^. The skin’s barrier property underwent notable modifications (*p* < 0.05) following incubation with CAGE and CAGE-P gel formulations, as seen in the biophysical investigations conducted at various time intervals. Taken together, CAGE-Pluronic F-127 ionogel is promising and efficient as a topical formulation for the administration of VH in a localized and systemic manner.

## Graphical abstract

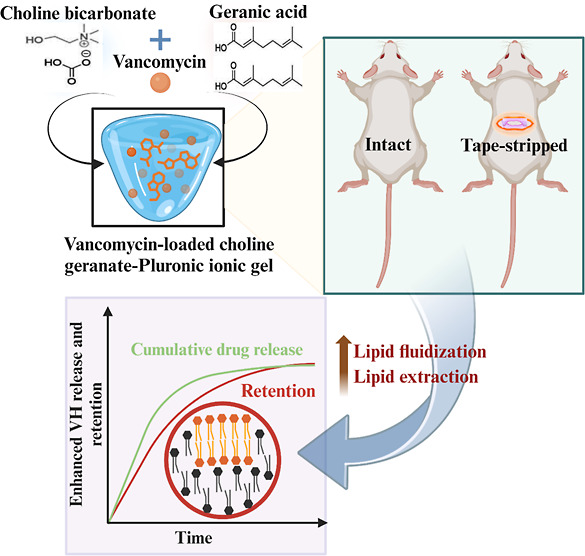

## 1 Introduction

Transdermal drug delivery systems have led to their acceptance as a practical, non-invasive approach to therapeutic administration due to benefits that include improved patient compliance, prolonged therapeutic effects, and evasion of first-pass metabolism, among others. Nonetheless, its widespread application is limited due to the skin’s remarkable impermeability. The stratum corneum (SC), the outermost layer of the epidermis, serves as an exceptional barrier against chemical absorption due to its distinctive hierarchical structure composed of numerous lipid bilayers and embedded corneocytes. A multitude of strategies have been employed to disrupt the skin barrier and improve transdermal drug delivery, encompassing physical disruption and chemical disruption, as well as hybrid approaches combining these techniques.^1,2^ To this end, in our previously reported work, we proved that the permeation of a model drug, vancomycin hydrochloride (VH), across the intact skin was not feasible owing to its high molecular weight (MW: 1486 Da) and high aqueous solubility (50 mg/mL). Moreover, the log *P* value of VH is reported to be −3.1. Topical administration is possible for the molecules with log *P* values between 1 and 4, which can permeate the lipophilic SC and enter the hydrophilic viable epidermis and dermis.^3^ Thus, oleic acid (OA), palmitic acid (PA), and anodal iontophoresis increased VH permeability across the intact and tape-stripped skin.^4^ In continuation of our previous work, we present the application of ionic liquids (ILs) and their effect on the improved permeability of VH across the SC and viable epidermis (after the removal of SC) using the tape-stripping technique.

ILs are organic salts made of ionic species that melt below 100 °C. Low vapor pressure, excellent conductivity, and a low melting point are just a few of the beneficial properties of ILs.^5,6^ For their one-of-a-kind characteristics, ILs have found applications as solubilizing agents capable of dissolving a broad variety of compounds and as permeation enhancers that can influence biological membranes to boost effectiveness and clinical results. ILs are mainly composed of two distinct components: cation and anion moieties. To this end, a mixture of cationic and anionic ILS has been reported to enhance the skin penetration of drugs. Among various types of ILs, choline bicarbonate (CB; cation) and geranate (GA; anion) have shown significant permeation for the model drugs, including acyclovir, bovine serum albumin, ovalbumin, and insulin, among others, by improving their solubility.^7–9^ These studies suggested the extraction of lipids from SC as one of the important mechanisms that facilitate the permeation of large hydrophilic molecules across the skin.^10,11^ Choline and some organic acids, such as malic, citric, and GAs, are generally recognized as safe and are extensively utilized as pharmaceutical excipients. The ILs of choline and GA has antimicrobial properties and enhanced transdermal transport of proteins and RNAi.^12,13^ Recently, the application of choline/OA (CO) and choline/malic acid in drug delivery systems was investigated for the transdermal delivery of antigen peptide and dextran (a model hydrophilic drug), which showed enhanced skin permeation and low skin irritation.^14,15^ These categories of ILs have been reported as biocompatible and less cytotoxic.^16,17^

Designing topical formulations, such as nanogels, hydrogels, organogels, bigels, and emulgels, among others, has recently attracted a great deal of attention. Semisolid preparations are believed to have advantages concerning the ease of application, drug solubilization, significant retention, and permeation within the skin. However, it is challenging to integrate all these characteristics into one semi-solid formulation. To this end, the preparation and application of organogels have been explored. Semisolid, bicontinuous organogels contain gelators and apolar solvents in three-dimensional network voids. Organogels can be translucent or turbid depending on the apolar solvent.^21^ Important organogel components that aid in drug permeation into the SC include terpenes, essential oils, surfactants, glycols, and fatty acids. These gel preparations are thermodynamically stable, non-toxic, and non-irritating.

Pluronic F-127 (Poloxamer 407; copolymer PEO_106_–PPO_70_–PEO_106_), a biocompatible, non-ionic surfactant, produces gels at 37 °C.^22^ Pluronic F-127 gel shows gel-to-sol transition at <25 °C.^23,24^ Monomolecular micelles are formed at concentrations of 10^–4^ to 10^–5^%, whereas multimolecular aggregates are produced at higher concentrations.^25,26^ Pluronic F-127 is frequently utilized as a gel matrix for the preparation of topical preparations containing various active therapeutics.^27–30^ Taken together, in the present study, different choline-based formulations were prepared, including the mixture of choline and OA (CO), choline and PA (CP), and choline and GA (CAGE) and were eventually characterized. Figure 1 depicts the chemical structure of these choline-based preparations. The optimized choline-based formulation was mixed with Pluronic F-127 to prepare Pluronic ionic gel-loaded with VH. Notably, the mechanistic investigation of these choline-based formulations on the lipids of SC or viable epidermis and their effect on the enhancement of dermal delivery of VH are further reported.

**Figure 1:**
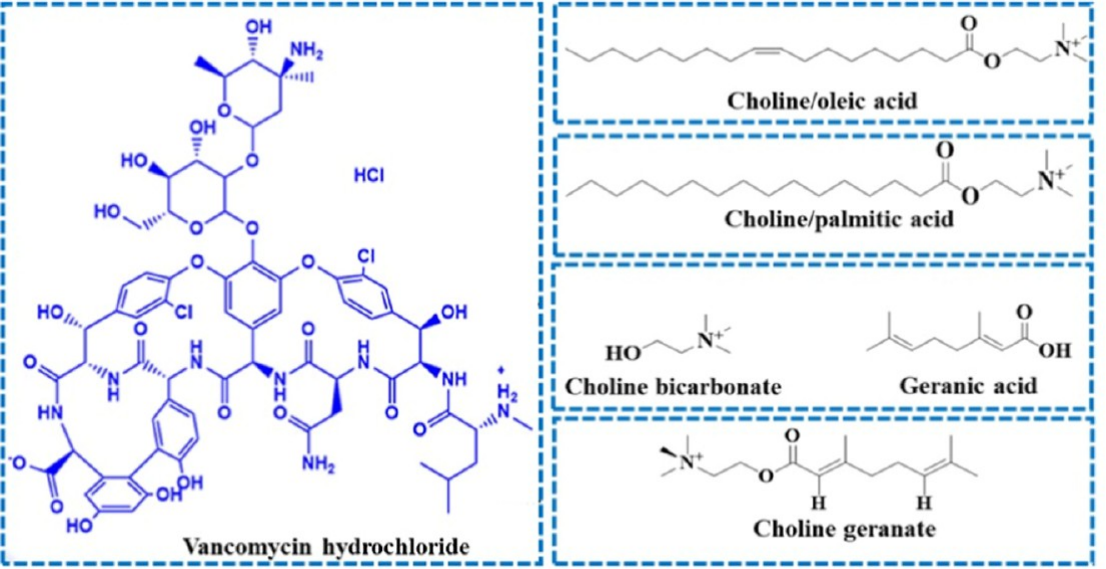
Chemical structures of VH, choline/OA (CO), choline/PA (CP), and choline geranate (CAGE).

## 2 Experimental Section

### 2.1 Materials and Methods

#### 2.1.1 Materials

Various chemicals were sourced from Sigma-Aldrich Chemical Company in Bengaluru, India, including pharmaceutical secondary standard VH, technical grade CB (80% in H_2_O), GA (85% in H_2_O), acetonitrile, and orthophosphoric acid. The analytical reagent grade PA and OA (99+%) were purchased from standard deviation (SD) Fine-Chemical Limited and Sisco Research Laboratories Pvt. Ltd, respectively. The adhesive tapes used for in vitro tape stripping were purchased from 3M in St. Paul, MN, USA. The tapes were 19 × 50 mm in size. Every experiment relied on Milli-Q water, manufactured by Millipore Inc. in the USA.

### 2.2 Preparation of VH-Loaded Choline/Oleic or PA and CAGE-ILs

The reaction schemes for the enhancers are shown in Figure S1. Two equivalents of 80 wt % choline bicarbonate (CB; 0.352 mL) and one equivalent of oleic acid (OA; 0.297 mL) or palmitic acid (PA; 0.256 g) were dissolved in phosphate buffer saline (PBS; pH 7.4)/isopropyl alcohol mixture in the optimized ratio of 1:1. To the mixture of CB and OA or CB and PA, a weighed amount of 32.45 or 32.63 mg of VH was added, respectively, to yield 50 mg/mL of VH concentration (Table 1). The solution was stirred at ambient temperature until carbon dioxide (CO_2_) ceased (Figure S1a).

**Table 1:** Different Choline-Based VH Formulations

components	choline/oleic acid	choline/palmitic acid	choline geranate (CAGE)
	% weight (w/v)		
oleic acid (OA)	0.25		
palmitic acid (PA)		0.25	
choline bicarbonate (CB)	9.4	9.4	9.4
geranic acid (GA)			21.5
vancomycin hydrochloride (VH)	5	5	5
phosphate-buffered saline (pH 7.4)	42.67	42.67	
isopropyl alcohol	42.67	42.67	

In an open round-bottom flask, one equivalent of 80 wt % solution choline bicarbonate (CB; 1.76 mL) was added to two equiv of 85 wt % GA (4.01 mL) to make the CB and GA combination. The mixture was agitated at room temperature until CO_2_ ceased. 292.2 mg of VH was added to the mixture to make a 50 mg/mL solution. To obtain a clear solution, the flask was stirred at room temperature for 1 h at 200 rpm (Figure S1b). The obtained solutions of choline/OA (CO), choline/PA (CP), and choline-geranate (CAGE) were used for physical characterization and skin permeation studies.

### 2.3 Characterization of Choline/Oleic or PA and CAGE-ILs

Nuclear magnetic resonance (NMR; AVANCE NEO 400 MHz, Bruker, USA) spectroscopy analysis was performed for the prepared formulations, including neat OA, PA, CO, CP, and CAGE. Samples were prepared by solubilizing 500 μL of these individual solutions (OA, CO, and CP) in 500 μL of deuterated dimethyl sulfoxide (DMSO). The PA solution was formulated by dissolving 5 mg in 1 mL of deuterated DMSO. Topspin 4.0.3 software was used to analyze the obtained spectra.

X-ray powder diffraction analysis (X-RPD; Ultima IV, Rigaku, Japan) was conducted for neat CO, CP, and CAGE to evaluate their crystalline or amorphous characteristics. A 1.542 Å copper Kα radiation source was used to scan the samples from 5 to 50° 2θ. At room temperature, the step size was 0.01° and the scanning speed was 1°/min.

Differential scanning calorimetry (DSC; DSC-60, Shimadzu, Japan) analysis was conducted for CO, CP, and CAGE. Samples were weighed in the range of 5–7 μL in the standard liquid aluminum pans. The pans were sealed, and the thermograms were documented following the heating of the samples at a rate of 5 °C/min from 28 °C–250 °C.

Fourier transform infrared (FT-IR; JASCO FT/IR 4200, Japan) spectroscopic analysis was performed for neat CO, CP, and CAGE. In brief, a moisture-free potassium bromide (KBr) pellet was used to drop-cast 20 μL of liquid sample from each solution, which was then dried for 5 min. Transmittance values were measured across the wavenumber range of 4000–400 cm^–1^.

### 2.4 Measurement of Zeta Potential and Conductivity

The choline-based formulations (CO, CP, and CAGE) were characterized for their charge and conductivity using a Malvern Zetasizer (Nano ZS; Malvern Instruments, UK). The measurements were conducted at a temperature of 25 °C. The samples were diluted with the mixture of PBS/IPA in the ratio 1:1, except for the CAGE solution. From these prepared samples, 1 mL was filled into folded capillary cells (DTS1070). Triplicate measurements were taken to determine the average zeta potential and conductivity.

### 2.5 Molecular Docking

Molecular docking analysis was conducted on a Schrodinger Suites 2022-1 (San Diego, CA). The docking preparation and binding affinity assessment for the choline-based formulations (CB, GA, CO, CP, and CAGE) with human keratin (Protein Data Bank (PDB) 3TNU) were conducted by using Auto Dock Vina screening software. The three-dimensional (3D) structures were acquired from the PDB, and a ligand library was generated.

### 2.6 High-Performance Liquid Chromatography Method to Analyze VH

A C8 column (150 × 4.6 mm; Phenomenex Inc., USA) with a pore size of 100 Å was used to optimize the high-performance liquid chromatography (HPLC) technique for analyzing VH. The mobile phases utilized were Milli-Q water (91.5% v/v acidified with 0.125% v/v orthophosphoric acid) and 8.5% v/v acetonitrile. At a flow rate of 0.5 mL/min, the acidified water and organic components were pumped in a 95:5 ratio. At a wavelength of 205 nm, the eluted VH was identified. The standard samples were made in a pH 7.4 solution of 100% PBS, and their concentrations varied between 0.5 and 50 μg/mL. It was determined that VH had a retention time of 3.6 min. The results of the regression study showed a correlation coefficient of 0.9999 and a regression equation of *y* = 26369*x* – 1191.2. The quantity of VH retained and penetrated within or across the skin samples was quantified in both in vitro and ex vivo investigations using the same HPLC technique.

### 2.7 Preparation of the Skin Samples

The freshly excised porcine ears were acquired from a nearby butcher. Under running water, the ears were cleaned. The underlying cartilage was detached from the dorsal skin using a knife and forceps. Using a dull scalpel, the hypodermis was carefully scraped away and the skin was examined closely for signs of damage. The skin samples were stored at a temperature of around 20 °C and used for not more than three months. This served as intact skin samples for release studies, as discussed later.

To improve the skin permeability of VH, the SC’s barrier properties were compromised by tape-stripping.^4^ In a nutshell, the skin samples were prepared by shaping circular discs of Scotch Book tape (845 3 M, St. Paul, USA) into an area of 0.7 cm^2^, and then, the tape was stripped off. Using finger pressure, the tape was applied to the skin and then removed quickly and evenly. Every single layer of SC was removed employing tape stripping. A bright field microscope (IX 53, Olympus Inc., Japan) was used to examine the tape strips. The 20× objective lens was used to visualize and examine the tape that was attached to a microscopic slide. Photos were taken with the charge-coupled device camera and the Q-capture PRO 7TM program. Furthermore, a bright field microscope was used to analyze the skin cross sections after SC removal. The skin samples were carefully placed in optimal cutting temperature medium before and after tape removal, and then frozen at −80 °C. Using a cryotome (Leica CM 1520, Leica Microsystems, Germany), the frozen tissue blocks were then cut into slices that were 6 μm thick. After that, the skin samples were dehydrated, stained with hematoxylin and eosin (H&E), and then examined under a bright-field microscope. Images were captured with a 10× objective lens.

### 2.8 Measurement of Transepidermal Water Loss and Skin Resistance

Transepidermal water loss (TEWL) and transepidermal electrical resistance (TEER) were used to ensure the integrity of skin samples. A vapometer (Delfin Technologies, Inc., Finland) was placed atop the donor chamber of a Franz diffusion cell to measure TEWL (g/m^2^/h). In the experiment, only skin samples that had TEWL values <10 g/m^2^/h were used. A continuous power supply unit was used to apply a direct current of 0.3 mA/cm2 (*I*) to evaluate the TEER (kΩ/cm^2^). After adding 0.2 mL of PBS solution to the donor chamber, the cathode was inserted into the receptor chamber with PBS, and the anode was introduced into the donor chamber. To find the potential difference (*V*) across the skin, a multimeter (Fluke 189, USA) was used. Skin samples were considered in the investigation only if their TEER values were more than 10 kΩ/cm^2^.

### 2.9 Ex Vivo Skin Permeation Studies

Using a Franz diffusion assembly, the skin penetration of VH was measured across the intact skin. The donor and receptor compartments were sandwiched with the excised porcine skin, which had a thickness of 0.38 to 0.40 mm. The capacity of the receptor compartment was 5 mL, and the exposed skin surface area was 0.64 cm^2^. 2 mL of a 50 mg/mL VH solution comprising three distinct choline-based formulations (CO, CP, and CAGE) was added to the SC that was sandwiched between the donor and receptor chambers. For the control formulation, 50 mg/mL of a VH solution in PBS (pH 7.4) was used. VH’s transdermal permeation was conducted for 48 h. At a temperature of 32 ± 1 °C and a stirring speed of 600 rpm, the conditions were ideal. At different intervals (0.5, 1, 2, 4, 6, 8, 12, 16, 24, 36, and 48 h), the same volume of PBS was substituted with 0.3 mL aliquots of the receptor medium to maintain the sink condition. The skin was kept at −20 °C until further use after 48 h of the experiment. Using the tape-stripping process, the viable skin was removed from the hydrated SC. Scotch Book tape (845, 3M, USA) was sequentially used five times to remove the treated skin area (0.64 cm^2^). These skin samples were analyzed in the same way as our previous research to determine the retention of VH within the skin samples.^4^

Time-dependent VH permeation across the skin was plotted. The slope of the regression line fitted to the linear component of the profile was used to determine transdermal drug flux using cumulative VH permeation data. The skin flux can be ascertained empirically by using the subsequent equation

1 $$J=\frac{\left(dQ/dt\right)}{A}$$

Where *J* represents the steady-state flux (μg/(cm^2^·h)), *A* is the diffusion area of skin tissue (cm^2^) involved in drug permeation, and d*Q*/d*t* is the quantity of drug traversing the skin per unit time at a steady-state (μg/h). The intercept of the extrapolated linear region with the *x*-axis yielded the lag time. The following equation was employed to determine the permeability coefficient (Kp)

2 $$Permeability\;coefficient\;\left(Kp\right)=\frac{Flux\;\left(F\right)}{Soluble\;concentration\;of\;VH\;in\;donor\;chamber\;\left(C\right)}$$

Skin permeation studies were also performed across tape-stripped skin samples. The SC was compromised, where the skin barrier was impaired using 12 tapes. The tapes were applied using gentle pressure onto the skin surface with the thumb, held for 3 s, and removed rapidly, applying minimal force. These tape-stripped skin samples were placed between the donor and the receptor chambers. For 48 h, these tape-stripped skin samples were treated with the formulations. The % recovery was estimated by comparing the peak regions of VH extracted from the skin samples, equivalent to known VH amounts.

### 2.10 Preparation of CAGE-Pluronic F-127 Ionogel

The best-optimized choline-based enhancer, based on ex vivo skin permeation studies, was carried out for the fabrication of the ionogel formulation. The ionogel was prepared by using Pluronic F-127 at a concentration of 22.7% w/v. The Pluronic F-127 and PEG-400 were mixed at 60 °C with constant stirring for 30 min. A separate beaker was used to mix 5.8 mL of CAGE and VH (292.2 mg) at room temperature for 1 h, as shown in Table 2. At 55 °C, the Pluronic F-127 gel base was mixed with the CAGE–VH mixture and stirred for 45 min (Figure S1c). The resulting gel (VH + CAGE-P ionogel) was then stored at 2–8 °C until further use.

**Table 2:** Optimized CAGE-Based VH Ionogel Formulation

components	% weight (w/v)
choline bicarbonate (CB)	9.4
geranic acid (GA)	21.5
vancomycin hydrochloride (VH)	5
pluronic F-127	22.7
PEG-400	41
^a^tea tree oil	0.4

a Tea tree oil was used as a fragrance to mask the odor of the gel formulation.

### 2.11 Characterization of VH-Loaded CAGE-Pluronic F-127 Ionogel

To check for phase separation, color, and clarity, the gels were inspected visually. The pH of the gels was measured using a digital pH meter (Eutech Instruments, Hyderabad, India).

To determine the crystallinity or amorphous properties of neat VH, Pluronic F-127, blank CAGE-P, and VH + CAGE-P gel, powder X-ray diffraction (XRD) analysis (PXRD, Ultima IV, Rigaku, Japan) was used. Using a 1.542 Å copper Kα radiation source, the samples were scanned from 5° to 50° 2θ. Scanning was done at ambient temperature with a step size of 0.01° and a speed of 1°/min.

Thermal transitions for the samples of neat VH, Pluronic F-127, VH in CAGE, blank CAGE-P, and VH + CAGE-P gel were studied using DSC. Solid samples or liquid samples were weighed between 5 and 7 mg or 5–7 μL. These weighed samples were placed in the aluminum sample holder. The samples were heated at a heating rate of 5 °C/min from 30 to 250 °C. The thermograms for each sample were recorded.

The neat VH, Pluronic F-127, blank CAGE-P, and VH + CAGE-P gels were all analyzed using FT-IR spectroscopy. To make a pellet, the solid samples were combined with dry KBr in a ratio of 1:100 and then compressed. From the 4000–400 cm^–1^ wavenumber, the percentage of transmittance was measured.

The VH + CAGE-P gel was micrographed at high resolution by using scanning electron microscopy (SEM). SEM images were captured at 500× and 2000× magnifications by using xT Microscope Control software. The samples were sputtered with a 10 nm layer of gold. The pictures were taken by using an accelerating voltage of 20 kV, a resolution of 1.5 nm, and a working distance of 10 mm.

The ionogel’s contact angle was measured with a Goniometer (Attension Theta Flex, Biolin Scientific, UK). Gel was applied to excised porcine ear skin. The automatic pipette applied 10 μL of gel solution to the skin using the sessile drop method. One Attension program measured contact angles at 0, 20, 40, and 60 s.

The Anton Par MCR 302, an Austrian modulated compact rheometer, was used for the rheological examination. To determine the linear viscoelastic properties, oscillatory experiments were performed, which included frequency, amplitude, and temperature sweep measurements. A 25 mm parallel plate–plate configuration (D-PP25 measuring system; P-PTD200+H-PTD120 measuring cell) was used to conduct amplitude sweep measurements. As the strain values increased from 0.01% to 1000%, the storage modulus, loss modulus, and complex viscosity were evaluated at room temperature with a constant rotational frequency of 10 rad/s. A temperature sweep test was performed, increasing the temperature by 2 °C per minute, from 25 to 60 °C. Changing the shear rate from 0.01 to 500 s^–1^ at 25 °C was used to measure viscosity.

Swelling studies were performed by suspending 0.3 g of gel in 5 mL of PBS, pH 7.4, at 37 °C. The swelling index of blank and VH-loaded CAGE-P gel was determined in glass Petri plates. Initial and final weight at different time intervals was recorded. Using eq 3, the swelling index was calculated.

3 $$Swelling\;index\;\left(\%\right)=\frac{\left(Final\;weight-Initial\;weight\right)}{Initial\;weight}\times 100$$

Porosity was measured to determine fluid penetration via the VH + CAGE-P gel. The solvent displacement method was used to measure the ionogel’s porosity. The solvent was absolute ethanol. The ionogel was carefully weighed and submerged in absolute ethanol for 0.5, 1, 2, and 4 h. After each time point, the swollen gel was removed, wiped with lint-free tissue paper, and weighed again. The formula computed the percent porosity change over time.

4 $$Porosity\;\left(\%\right)=\frac{\left(V_{1}-V_{3}\right)}{\left(V_{2}-V_{3}\right)}\times 100$$

Where *V*_1_ indicates the initial volume of the absolute ethanol, *V*_2_ indicates the total volume of the solvent with the immersed gel, and *V*_3_ indicates the volume of the solvent after removal of the gel sample.

### 2.12 In Vitro VH Release Kinetics

To assess the release of VH or VH from CAGE and CAGE-P gels, Franz diffusion cells were used. The donor and receptor compartments were connected by dialysis tubing (Himedia, India, with a molecular weight cutoff of 13 kDa). A 0.2 mL of neat VH, VH + CAGE, or VH + CAGE-P gel formulation was spiked onto the donor chamber. All of these formulations included 50 mg/mL of VH. Receptor media contained 7.4 pH fresh PBS. At a temperature of 32 ± 1 °C and a stirring speed of 600 rpm, the conditions were ideal. At different intervals (0.5, 1, 2, 4, 6, 8, 12, 16, 24, 36, and 48 h), the same volume of PBS was substituted with 0.3 mL aliquots of receptor medium to maintain the sink condition. The following equations compute the percentage of VH released

5 $$Release\;of\;VH\;\left(\%\right)=\frac{cumulative\;amount\;of\;VH}{concentration\;of\;VH\;used}\times 100$$

The following equations were used to model the drug release profiles

6 $$Zero‐order\;release\;model;\;F=K_{0}t$$

Where *F* is the fraction of VH released at time *t*, and *K*_0_ is the zero-order rate constant.

7 $$First‐order\;release\;model;\;In\left(1-F\right)=K_{1}t$$

Where *K*_1_ is the first-order rate constant.

8 $$Higuchi\;model;\;F={-K}_{H}t^{1/2}$$

Where *K*_H_ is the Higuchi rate constant.

9 $$Korsmeyer‐Peppas\;model;\;\left(M_{t}/M_{\infty }\right)=K_{kp}t^{n}$$

Where (*M*_*t*_/*M*_∞_) is the fraction of VH released at time *t*, *K*_kp_ is the release rate constant, and *n* is the release exponent. The estimated “*n*” value was used to categorize the various release mechanisms. Quasi-Fickian diffusion is *n* < 0.5, Fickian diffusion is *n* = 0.5, non-Fickian diffusion is 0.45 < *n* < 1, case-II transport is *n* = 1, and super case-II transport is *n* > 1 [40].

### 2.13 Ex Vivo Skin Permeation Studies

The skin permeation of VH across the intact and tape-stripped skin was assessed by using a Franz diffusion assembly. The excised porcine skin, measuring 0.38–0.40 mm in thickness for intact skin and 0.28–0.30 mm for tape-stripped skin, was positioned between the donor and receptor compartments. These skin samples were treated with 0.2 mL of neat VH solution (50 mg/mL) as a control and VH + CAGE-P gel (5% w/w) formulation. The penetration of VH across the skin was studied for 48 h. To maintain the sink condition, 0.3 mL aliquots of receptor medium were withdrawn and replaced with the same volume of PBS at 0.5, 1, 2, 4, 6, 8, 12, 16, 24, 36, and 48 h. After 48 h of experiment, these samples were analyzed using an optimized HPLC technique and various skin permeation parameters including flux (μg/(cm^2^·h)), lag time (h), the cumulative amount of VH permeated (μg/cm^2^), diffusion coefficient (cm^2^/h), and permeability coefficient (cm/h) were calculated using the mathematical formulas described earlier (Section 2.9). Later, SC and viable skin samples were processed to quantify the amount of VH retained, using the method described in our previously reported work.^4^

### 2.14 Mechanistic Studies to Determine the Action of Choline-Based ILs Enhancers

Mechanistic studies, including SEM, FT-IR spectroscopy, and DSC thermal analysis, were performed for both SC and viable epidermal samples to understand the action of choline-based ILs enhancers.

SC and viable skin samples treated with 0.2 mL of CO and CP in a 2:1 molar ratio, dissolved in a combination of 50% PBS/IPA solution, were visualized for high-resolution micrographs using SEM. In an additional series of trials, skin samples were exposed to 0.2 mL of CAGE and CAGE-P gel (0.2% w/w) formulations with a molar ratio of 1:2. These skin samples were treated at two different time points, i.e., 2 and 12 h. Using the xT Microscope Control program, scanning electron micrographs were taken at magnifications ranging from 500× to 2000×. The SC and viable epidermal skin samples were sputter-coated with 10 nm of gold. The photos were taken at an accelerating voltage of 20 kV and a resolution of 1.5 nm.

At predetermined intervals, FT-IR analysis was carried out for the SC and viable skin samples that had been treated with various formulations. The viable skin was subjected to 60 °C for 90 s in warm water to remove the SC (Figure S6). Skin samples (SC or viable skin) were placed on a Franz diffusion cell at a temperature of 32 ± 1 °C. A 2:1 molar ratio of CO and CP, dissolved in a 50% PBS/IPA solution, was applied to the excised SC and viable skin for 2 and 12 h, respectively. For the same durations in an alternative set of trials, 0.2 mL of CAGE in a 1:2 molar ratio and 0.2% w/w CAGE-P gel were used. Following 2 and 12 h, the samples were washed with 3 mL of PBS solution, blotted dry with lint-free Kim wipes, and subsequently left to air dry for 1 h at room temperature. Potassium bromide was mixed with the dried samples in a 1:100 ratio. The samples were then compressed into a pellet with a force of 40 kN. A 50% combination of PBS and IPA was applied to the SC and viable skin samples for the same time as a control. In the 4000–400 cm^–1^ wavenumber region, the spectra were obtained by using FT-IR with a 4 cm^–1^ resolution.

For DSC studies, a typical 40 μL aluminum pan was filled with 2–5 mg of separated SC or viable epidermal skin sheets. For 2 and 12 h, the samples were exposed to 0.2 mL of CO and CP in a 2:1 molar ratio and dissolved in 50% PBS/IPA solution. The skin samples were also exposed to 0.2 mL of CAGE (1:2 molar ratio) and CAGE-P gel (0.2%, w/w) for a similar time. The skin samples treated with a 50% PBS/IPA mixture served as the control. Thermal analysis was done with a Shimadzu DTG-60. Under nitrogen flow, all samples were heated to 28–200 °C at 2 °C per minute. The transition temperatures and enthalpy changes were recorded.

### 2.15 In Vivo Skin Irritation Analysis

The study utilized male Sprague-Dawley (SD) rats, aged 8–10 weeks, with an average weight of 270 ± 20 g. The animal experimentation procedure received approval from the Institutional Animal Ethics Committee (IAEC) at BITS Pilani, Hyderabad Campus (HYD/IAEC/2021/09). Separate cages were utilized to house the various animal groups, and a combination of 5% isoflurane and 21% oxygen was pumped at a flow rate of 1 L/min to put them to sleep (EZ Animal Systems, Canada). The 3 cm^2^ of dorsal surface hair was delicately and painlessly trimmed using a hair clipper. Group 1 was considered as a control with healthy rats without tape-stripping of the SC. Book tape (845, Scotch book tape, 3 M, USA) was used to tape-strip the SC for three separate groups. The SC was entirely removed using a total of 10 strips. After 24 h of tape-stripping, 10 μL of the final optimized enhancer (CAGE-IL) or formulation (VH + CAGE-P gel) was uniformly applied. Skin blood flow was evaluated using Laser Doppler flowmetry (LDF; MoorLDI2-IR, Moor Instruments Ltd., Devon, UK). The LDF employed a helium–neon red gas laser with a wavelength of 635 nm. The scanned region measured 3 cm^2^, with a scan duration of 4 ms per pixel for 1 min. The data were evaluated offline utilizing the manufacturer’s software suite. The flow rate was recorded for 1 min under anesthesia. The application of LDF for evaluating skin irritation reactions was as per the ESCD standard.^31^ Moreover, TEWL values were measured to understand the skin’s integrity. A ventilated chamber was used to measure TEWL, and the temperature and relative humidity were regulated to be 25–28 °C and 38–40%, respectively. The skin irritation severity was determined as the mean ± SD values from epidermal blood flow in arbitrary units for the ROI and TEWL.

The animals were euthanized via cervical dislocation on 14th day. The dorsal skin was removed and kept at a temperature of −80 °C. To embed the skin sample in an optimum cutting temperature (PolyFreeze, Polysciences, Inc., USA), it was kept at −80 °C for 12 h. A cryotome (CM1520, Leica Biosystems, Germany) was used to slice the skin specimen into 5 μm-thick slices. For histological evaluation, H&E staining was performed. The slices were examined under a microscope (Olympus IX53, Japan, Olympus Corporation) to capture their microscopic images.

### 2.16 Statistical Analysis

Data are expressed as mean ± SD for all the experiments, repeated at least four times. An unpaired *t*-test (GraphPad Prism, version 5.03) was used to examine the differences between different treatment groups, where the level of significance difference was considered when *p* < 0.05.

## 3 Results

### 3.1 Characterization of CO, CP, and CAGE

The prepared mixture of choline/OA or PA turns from a clear to a whitish solution in the presence of CB. For the preparation of CAGE-ILs, the color of the solution was observed to be pale yellow, which can be attributed to the inherent yellow color of GA (Figure S2). However, all of these formulations were found to be clear with no particulates before or after the addition of VH. The pH for these formulations ranged between 5.20 and 5.50.

Figure 2a shows ^1^H NMR signals of neat or a mixture of permeation enhancers. The NMR spectra of choline showed methylene groups (−CH_2_) appearing at 3.8 (a) and 3.3 ppm (b). The methyl signal of the nitrogen atom was found to appear at 3.0 ppm (c). GA showed characteristic hydrogen signals at 5.1 ppm (c) and 5.72 ppm (f). Also, a small signal appearing at 11.5 ppm confirms the presence of the (−COOH) group in the GA molecule. PA and OA showed the presence of the (−COOH) group (labeled as d and e) at 12.0 ppm. However, the mixture of choline and OA or PA did not show any signal at 12.0 ppm, confirming the esterification reaction and resulting in the formation of choline/OA (CO) or choline/PA (CP). NMR spectra also showed the appearance of the methyl groups (−CH_3_) attached to the nitrogen of the choline molecule at 3.0 and 4.2 ppm for CP (d) and CO (d), respectively. The reaction between the choline and GA (CAGE-IL) showed the presence of methyl groups attached to the nitrogen atom of the choline molecule (a) at 3.1 ppm. Also, the protons of the hydroxyl chain of the choline (b and g) were observed at 3.4 and 3.8 ppm, respectively.^32^ The hydrogen signals (c and f) from the GA were observed at 5.6 and 5.0 ppm, respectively. The internal (−CH_3_) and (−CH_2_) groups (labeled as d and e) from GA were found to be at 1.5–1.7 and 2.0 ppm, respectively.^33^

**Figure 2:**
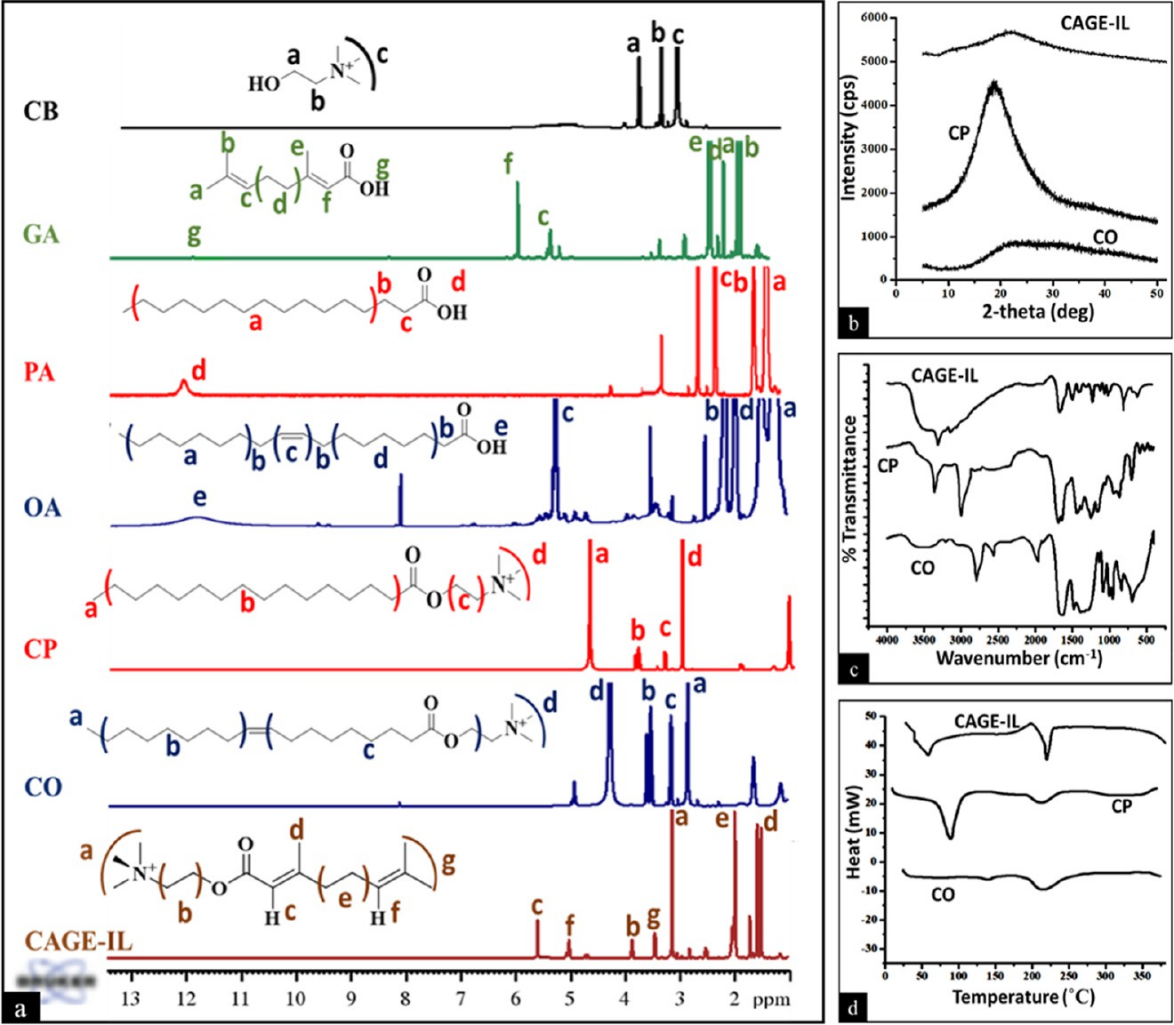
Representative spectra for characterization of neat or mixture of choline-based enhancers using ^1^H NMR spectroscopy (a), XRD (b), FT-IR spectroscopy (c), and DSC thermograms (d). CB—choline bicarbonate; GA—geranic acid; PA—palmitic acid; OA—oleic acid; CP—choline/palmitic acid; CO—choline/oleic acid; CAGE-IL—choline geranate ionic liquid.

The XRD pattern of CO, CP, and CAGE-IL formulations is represented in Figure 2b. All the formulations were found to be amorphous due to the absence of any sharp diffraction peaks at 2θ values of 5° to 50°.

Figure 2c shows the FT-IR spectra of the CO, CP, and CAGE-IL formulations. CO formulation has vibrational bands at 2854 cm^–1^ due to symmetric CH_2_ stretching. C=O and C–O stretching causes absorption bands at 1710 cm^–1^ and 1285 cm^–1^. The asymmetric CH_2_ band gave the CP formulation an absorption peak at 2924 cm^–1^. The presence of free COO- in PA caused distinctive peaks at 1640 cm^–1^.^34^ Due to C–O stretching, CAGE-IL had absorption peaks at 1105 and 1042 cm^–1^. C–N stretching was verified by absorption peaks at 1255 and 1323 cm^–1^. An absorption peak at 1640 cm^–1^ indicated C=O stretching. Intermolecular hydrogen bonding was indicated by broader and less intense peaks at 3100 and 3400 cm^–1^.

DSC thermal analysis was performed for the recognition of endothermic peaks for CO, CP, and CAGE-IL formulations (Figure 2d). The appearance of broad endothermic peaks at 210 °C in both CO and CP formulations is attributed to the interaction of CB with oleic and PA. The presence of a sharp endothermic peak at 71.2 °C in the CP formulation was due to the melting point of PA. The endothermic peak at 241 °C was due to the interaction of GA with CB.

### 3.2 Measurement of Zeta Potential and Conductivity

Figure S3 shows the zeta potential and conductivity measurements for neat CO, CP, and CAGE-IL formulations. All the formulations exhibited negative zeta potential. The surface charge of CO and CP formulations was −3.01 ± 0.8 mV and −28.39 ± 3.67 mV, respectively. However, the charge exhibited by the CAGE-IL formulation was significantly (*p* < 0.05) less compared to the CO and CP formulations. Similarly, the conductivity of CAGE-IL formulation was found to be significantly (*p* < 0.05) less in comparison to CO (24.43 ± 3.48 mS/cm) and CP (27.77 ± 2.49 mS/cm) formulations.

### 3.3 Molecular Docking

Since keratin is considered the most important component of skin lipids, 3TNU keratin was selected as the skin bilayer model. The enhancers (CO, CP, CB, GA, and CAGE) were docked against the keratin, and Figure S4 shows the interaction strength that was characterized using molecular docking. The inset table with the captured images indicates that all of the selected enhancers could form hydrogen bonds. The data showed that the strength of the hydrogen bond of CB (−3.335 kcal/mol) and CAGE (−3.170 kcal/mol) was the strongest, followed by those of CO, GA, and CP. Therefore, CB and CAGE showed strong hydrogen bond interactions or the highest interaction strength with keratin.

### 3.4 Preparation of Skin Samples

Porcine ear skin employed in ex vivo skin permeation studies had an average thickness of 0.383 ± 0.011 mm. Representative digital photos of intact skin and skin samples that were tape-stripped 12 times to completely remove SC are shown in Figure S5a. Concerning the tape stripping procedure, a greater quantity of corneocytes was removed with an increase in the subsequent stripping. Notably, the 12th strip showed a reduced distribution or adherence of corneocytes (Figure S5b). The histological photomicrographs that were captured showed the use of 12 tape-stripping can remove SC completely, and it was compared with the H&E-stained image of intact skin (Figure S5c,d). The average TEWL for intact excised porcine ear skin was determined to be 10.4 ± 2.14 g/m^2^/h, which increased to 40.17 ± 2.23 g/m^2^/h after 12 tape-strips. Conversely, the TEER decreased to 6.20 ± 2.31 kΩ/cm^2^ from 17.27 ± 1.75 kΩ/cm^2^. Both changes were statistically significant (*p* < 0.05).

### 3.5 Ex Vivo Skin Permeation Studies

The average thicknesses of intact and tape-stripped porcine ear skin employed in ex vivo skin permeation investigations were 0.39 ± 0.011 and 0.29 ± 0.015 mm, respectively. The TEWL and TEER values were measured throughout the experiment for skin samples treated with different formulations containing VH at different time intervals. In general, the TEWL and TEER values were found to increase and decrease with an increase in the treatment time (Figure 3a,b). Similarly, the same trend was observed for the tape-stripped skin (Figure 3c,d). The TEWL values for intact skin and tape-stripped skin treated with CAGE-IL formulation were found to be 44.8 ± 3.2 and 46.7 ± 2.2 g/m^2^/h, respectively, after 48 h of treatment.

**Figure 3:**
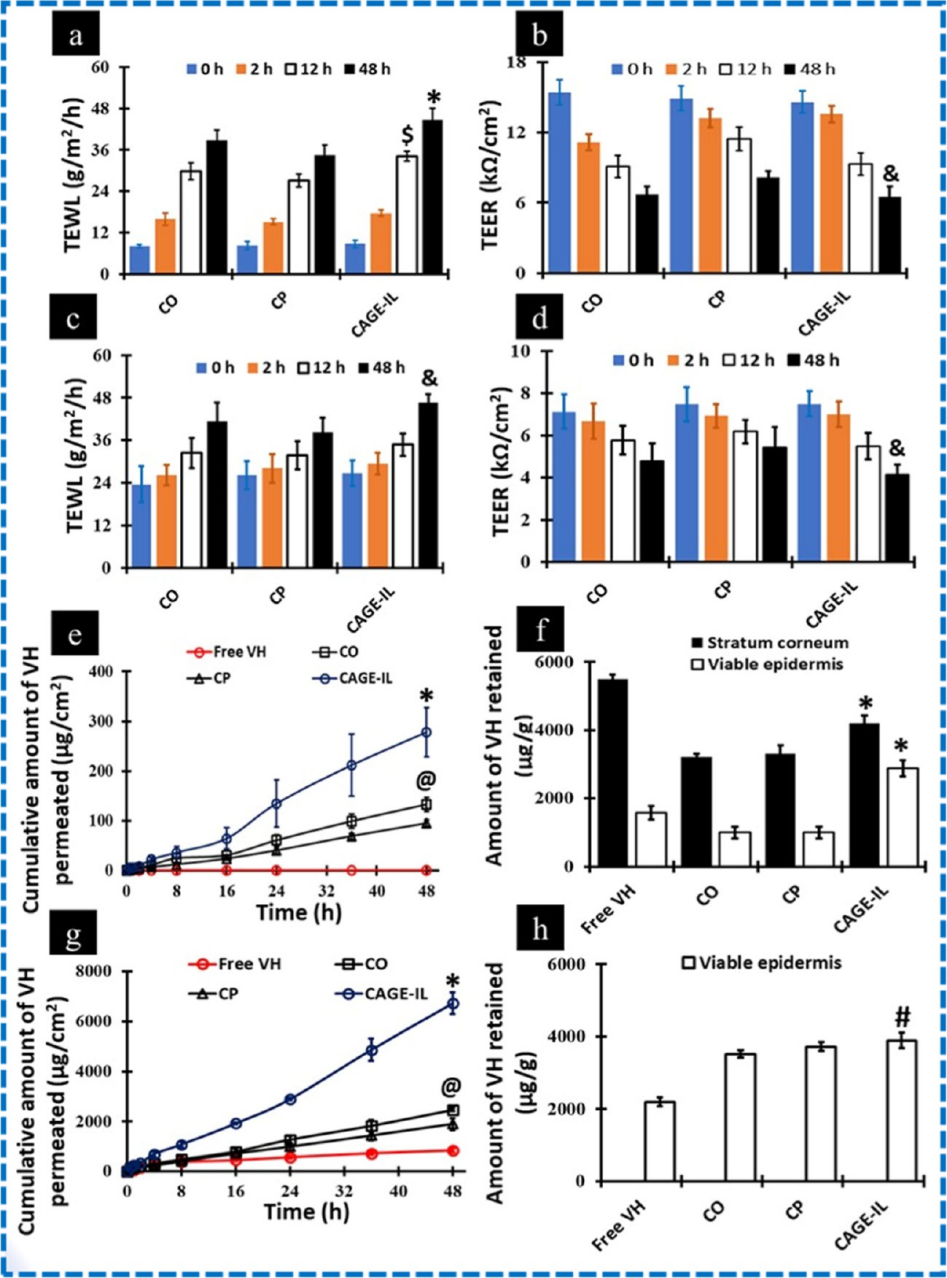
Measurement of TEWL and TEER in intact (a,b) and tape-stripped skin (c,d). Ex vivo skin permeation profile and retention of vancomycin across and within the intact (e,f) and tape-stripped skin (g,h). Values represent mean ± SD (*n* = 4). “*” denotes that the value is significantly different at *p* < 0.05 compared to all the other groups at 48 h. “$” denotes that the value is significantly different at *p* < 0.05 compared to all the other groups at 12 h. “&” denotes that the value is significantly different at *p* < 0.05 compared to the CP group at 48 h. “@” denotes that the value is significantly different at *p* < 0.05 compared to the CP group at 48 h. “#” denotes that the value is significantly different at *p* < 0.05 compared to all the other groups, except CP. CO—choline/oleic acid; CP—choline/palmitic acid; CAGE-IL—choline geranate ionic liquid.

Results showed that the TEER values for intact skin (6.5 ± 0.9 kΩ/cm^2^) and tape-stripped skin (4.2 ± 0.4 kΩ/cm^2^) treated with the CAGE-IL formulation, respectively, were obtained. VH extracted from the skin samples had a retention time of 3.6 min, which was similar to that of neat VH. A value of 0.75 μg/mL was determined to be the LOQ. The proportion of VH recovered from the excised porcine skin ranged from 82% to 88%. According to ex vivo skin permeation studies, neat VH did not permeate across the intact skin for up to 48 h (Figure 3e). Figure 3f shows that the amount of VH retained in viable skin was 1575 ± 201 μg/g, whereas in the SC it was 5504 ± 122 μg/g (Figure 3f). The permeation of VH was increased for the skin treated with CO, CP, and CAGE-IL formulations. Skin permeation was enhanced significantly for the CAGE-IL formulation (278 ± 49 μg/cm^2^), followed by the CO formulation (133 ± 14 μg/cm^2^) when compared with the skin samples treated with the CP formulation (95 ± 6 μg/cm^2^). VH penetration and retention across the tape-stripped skin are shown in Figure 3g,h. Permeation of neat VH across the skin was 838 ± 71 μg/cm^2^. However, the transport of VH increased significantly for the CAGE-IL formulation, which was found to be 6729 ± 437 μg/cm^2^. The amount of VH retained in the SC was found to be 3892 ± 215 μg/g after 48 h.

Table 3 shows the permeation parameters of VH transport across the skin. In intact skin, after the application of VH in the CO and CP formulation, the flux was found to be 2.72 ± 0.3 and 1.92 ± 0.1 μg/cm^2^/h, respectively. However, these values were significantly increased in the tape-stripped skin. The highest flux was found for the CAGE-IL formulation in both intact (5.80 ± 1.3 μg/cm^2^/h) and tape-stripped skin (134.30 ± 8.3 μg/cm^2^/h). The lag time for VH in CO and CP formulation for intact skin was found to be 0.30 ± 0.1 h and 0.67 ± 0.2 h, respectively. However, for the tape-stripped skin, the lag time for all the formulations was found to be zero. In comparison to the application of formulations including VH + CO and VH + CP, VH + CAGE-IL showed the highest cumulative amount released in both intact and tape-stripped skin (Table 3). Therefore, CAGE-IL was taken forward to be incorporated with Pluronic F-127 to prepare the VH-loaded ionogel formulation.

**Table 3:** Skin Permeation Parameters of VH after Treatment with Different Formulations^a^

Treatment groups	*T*_lag_ (h)	*J* (μg/cm^2^/h)	*K*_p_ (×10^–3^ cm/h)	*D* (×10^–3^ cm/h)	*Q*_48_ (μg/cm^2^)
intact skin					
untreated skin					
CO	0.30 ± 0.1	2.72 ± 0.3	0.27 ± 0.04	1.28 ± 1.1	133 ± 14
CP	0.67 ± 0.2	1.92 ± 0.1	0.18 ± 0.01	0.57 ± 0.22	95 ± 6
CAGE-IL	0.84 ± 0.4	5.80 ± 1.3^a^	0.63 ± 0.11	0.33 ± 0.03	278 ± 49^b^
tape-stripped skin					
untreated skin	9.3 ± 2.7	15.71 ± 1.3	1.5 ± 0.1	0.02 ± 0.004	838 ± 71
CO	0	49.30 ± 3.7	4.9 ± 0.3	0	2466 ± 62
CP	0	38.30 ± 5.2	3.8 ± 0.5	0	1902 ± 244
CAGE-IL	0	134.30 ± 8.3^a^	13.4 ± 0.8	0	6729 ± 437^b^

a *T*_lag_, lag time; *J*, Flux; *K*_p_, permeability coefficient; *D*, diffusivity; *Q*_48_, the cumulative amount of VH permeated after 48 h per unit area. The thickness of the intact and tape-stripped skin ranged from 0.38–0.40 mm and 0.28–0.30 mm, respectively. Data represent mean ± SD (*n* = 4). Untreated skin was served as a control, treated with neat VH dissolved in PBS solution. “a” and “b” denote that the flux values and cumulative amount permeated are significantly different at (*p* < 0.05) for both intact and tape-stripped skin compared to other formulation groups. CO—choline/oleic acid; CP—choline/palmitic acid; CAGE-IL—choline geranate ionic liquids; CAGE-P gel—choline geranate Pluronic ionogel.

### 3.6 Characterization of VH-Loaded CAGE-Pluronic F-127 Ionogel

The digital photographs of the fabricated VH + CAGE-P gel formulation are displayed in Figure 4a. The addition of CAGE-IL caused the formerly clear solution to become yellow. The VH + CAGE-P gel was observed to be completely free of particles and to have a homogenous consistency. The blank CAGE-P gel had a pH of 5.62 ± 1.21, whereas the VH + CAGE-P gel had a pH of 5.81 ± 0.78.

**Figure 4:**
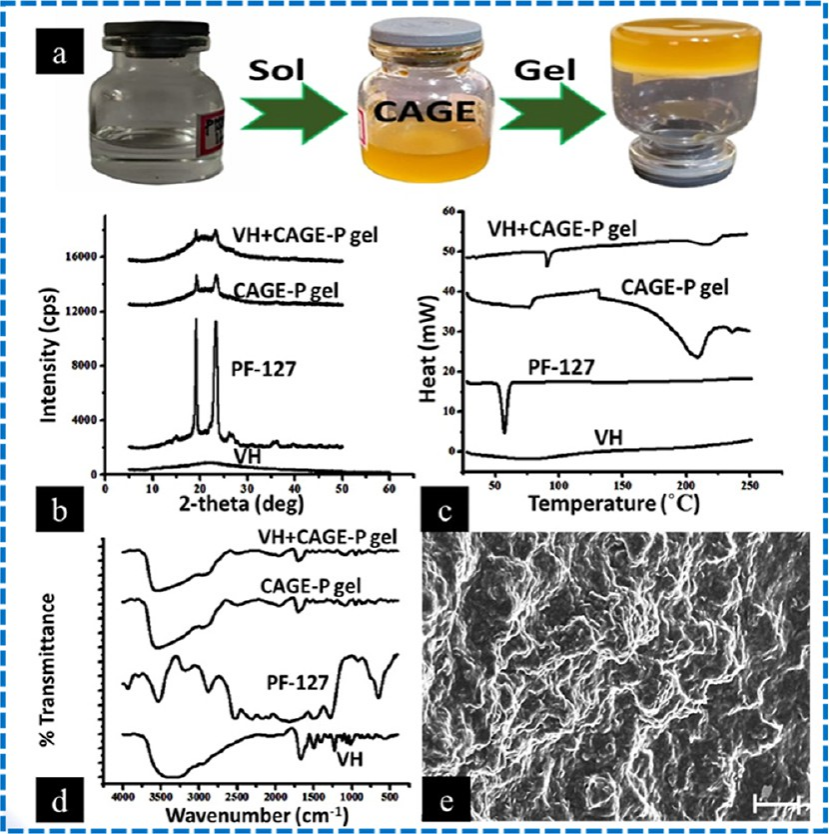
Digital images captured for the sol-to-gel transformation of VH + CAGE-P ionogel formulation (a). Representative powder X-ray diffractograms (b), DSC thermograms (c), and FT-IR spectra (d) of neat VH, Pluronic F-127, CAGE-P ionogel, and VH + CAGE-P ionogel. Scanning electron microscopic images captured for VH + CAGE-P gel formulation (e). The scale bar represents 100 μm.

Figure 4b displays the XRD patterns of pure VH, Pluronic F-127, blank CAGE-P, and the VH + CAGE-P gel. The VH was shown to be in an amorphous form without diffraction peaks. The X-ray diffractogram of pure Pluronic F-127 exhibited prominent peaks at 2θ values of 18.89 and 23.09°. The blank CAGE-P gel and VH + CAGE-P gel formulations exhibited peaks of diminished intensity at 2θ values of 18.89 and 23.09°, akin to Pluronic F-127. No distinctive diffraction peaks of VH were seen in the VH + CAGE-P gel composition.

Figure 4c displays DSC thermograms of neat VH, Pluronic F-127, blank CAGE-P, and VH + CAGE-P gel. The endothermic peak of neat VH and Pluronic F-127 was seen at 83.7 and 57.3 °C, respectively, identical to the reported melting points. The endothermic peaks at 220 °C in blank and VH + CAGE-P gels are due to CAGE interaction in the gel phase. Endothermic peak shifts to 73 °C for VH + CAGE-P gel, compared to 57.3 °C for neat Pluronic F-127, likely due to interaction in the gel matrix.

Figure 4d shows sample FT-IR spectra of neat VH, Pluronic F-127, blank CAGE-P, and the VH + CAGE-P gel. VH’s spectra exhibited distinctive peaks at 3406 cm^–1^ for hydroxyl stretching, 1658 cm^–1^ for C=O stretching, and 1230 cm^–1^ for phenolic hydroxy groups. Sharp bands at 3485 cm^–1^ caused a –OH group stretching vibration in Pluronic F-127 spectra. Intermolecular hydrogen bonding is shown by broader peaks at 3100 and 3600 cm^–1^ in the blank and VH + CAGE-P gels. The distinctive C=O peak at 1658 cm^–1^ indicates VH loading in the CAGE-P gel. The scanning electron microscopic image of VH + CAGE-P gel formulation is shown in Figure 4e. The prepared gel has a fibrous surface.

The experimental setup for measurement of the contact angle is depicted in Figure 5a. The excised skin from the porcine ear served as the experimental substrate. The contact angle changed over time, as shown in Figure 5b. The skin sample had a contact angle <50° when subjected to the VH + CAGE-P gel formulation.

**Figure 5:**
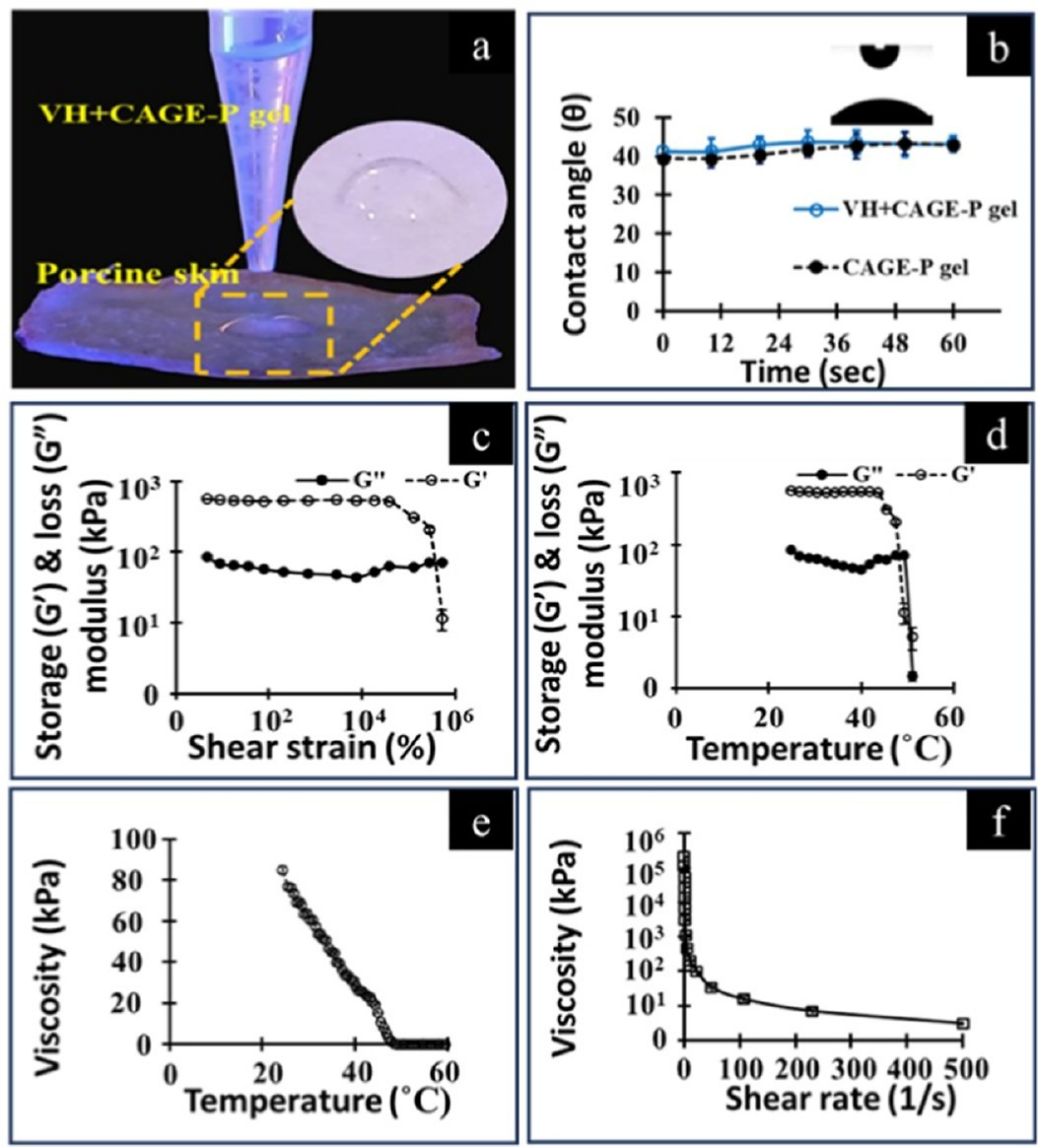
Experimental set-up for the measurement of the contact angle of ionogel on the skin using a goniometer (a). Measurement of contact angle with time (b). Rheological analysis of VH + CAGE-P ionogel formulation. Storage (*G*′) and loss (*G*″) moduli were determined in amplitude (c) and temperature sweep (d) studies. Change in viscosity with the increase in temperature (e) and varying shear rate (f). Data represents a mean of 3–4 repetitions. Refer to Table 1 for formulation composition.

The rheological parameters, including storage (*G*′) and loss (*G*″) moduli and viscosity (η), were determined for the VH + CAGE-P gel formulation. The gel formulation’s linear viscoelastic region is shown in Figure 5c. All subsequent rheological investigations maintained a shear strain of 1%. The Pluronic gel with VH had a gel-to-sol transition temperature of 45.8 ± 1.3̊C, as shown in Figure 5d. The storage modulus was greater than the loss modulus, indicating the gel phase below 40 °C. Figure 5e illustrates that the gel formulation has a shear-thinning property since the viscosity decreases as the temperature increases. As seen in Figure 5f, the viscosity of the VH + CAGE-P gel decreases as the shear rate increases.

To comprehend the behavior of the gel system in terms of solvent absorption, the swelling index was calculated in phosphate buffer saline, with a pH of 7.4, at 37 °C. Figure S7a demonstrates that the swelling index of the VH + CAGE-P gel formulation increases from 0 to 6 h of incubation. Nevertheless, the gel matrix began to dissolve after 6 h. The blank CAGE-P gel had a maximum swelling index of 32.21 ± 0.97%, whereas the VH + CAGE-P gel formulation had an index of 31.75 ± 1.06%. The experimental setup for the gel immersed in the medium is shown in the inset of the obtained image.

Figure S7b shows the increase in the percentage of porosity of the VH + CAGE-P gel formulation. The digital inset of the image captured indicates the experimental setup for the gel immersed in the organic solvent, ethanol, in a glass vial. The maximum percentage porosity of the blank CAGE-P gel and VH + CAGE-P gel formulations was found to be 71.53 ± 1.20% and 73.61 ± 2.52%, respectively. Scanning electron microscopic images were captured for the swollen gel in aqueous buffer media and organic solvent at different time intervals (2, 4, and 6 h). The increase in pores was observed with an increase in the incubation time from 2 to 6 h.

### 3.7 In Vitro VH Release Kinetics

CAGE and CAGE-P gels released a percentage cumulative amount of VH across the dialysis membrane (Figure 6). In vitro investigations revealed a burst release of neat VH, releasing 65.75 ± 10.42% in 4 h. However, the complete release of VH was achieved within 12 h. The percentage of VH released from the CAGE formulation was found to be 80.82 ± 5.80%. The percentage of VH diffused from the CAGE-P gel formulation was achieved slowly. The cumulative amount of VH released at 12 h was found to be 22.76 ± 0.63%, followed by 48.73 ± 0.72% at the end of 48 h.

**Figure 6:**
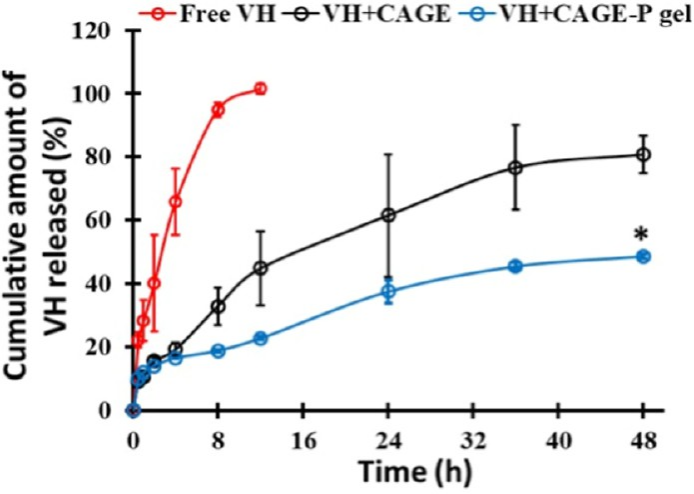
In vitro release profile of VH from different formulations. Data represent mean ± SD (*n* = 3). “*” indicates that the value is significantly different at *p* < 0.05 compared to other groups at 48 h.

Higuchi’s equation, Korsmeyer Peppas equation, first order, and zero order release kinetic models were used to examine in vitro VH release data. The VH release kinetic model with the greatest correlation (*R* ^2^) coefficient was the best. The optimized VH + CAGE-P ionogel formulation had the highest Higuchi model correlation coefficient (*R* ^2^ = 0977), defining VH release as a time-dependent diffusion process based on Fick’s law. Since the cumulative VH release was <60%, the Korsmeyer-Peppas model was not suitable for calculating release behaviour.^35,36^ In conclusion, VH was released by pure diffusion without matrix erosion or swelling (Table 4).

**Table 4:** Kinetics of In Vitro Release of VH From CAGE-P Ionogel Formulation^a^

kinetic models	VH + CAGE-P ionogel
zero-order	
*R* ^2^	0.966
*K*_0_	0.851
first-order	
*R* ^2^	0.897
*K*_1_	0.014
Higuchi	
*R* ^2^	0.977
*K*_H_	6.536

a *R* ^2^, regression coefficient; *K*_0_, zero-order rate constant; *K*_1_, first-order rate constant; *K*_H_, Higuchi constant.

### 3.8 Ex Vivo Skin Permeation Studies

The average thickness of the intact and tape-stripped excised porcine ear skin used for ex vivo skin permeation studies was 0.38 ± 0.011 and 0.29 ± 0.015 mm, respectively. The TEWL and TEER values were measured at 0, 2, 12, and 48 h after the application of VH + CAGE-P gel formulation in both intact and tape-stripped skin (Figure 7a,b). In intact skin, the TEWL and TEER were found to be 8.025 ± 0.43 g/m^2^/h and 17.32 ± 1.27 kΩ/cm^2^, respectively, at the initial zero time point. A significant increase in the TEWL and decrease in the TEER were found at the 48th h time point in both intact and tape-stripped skin.

**Figure 7:**
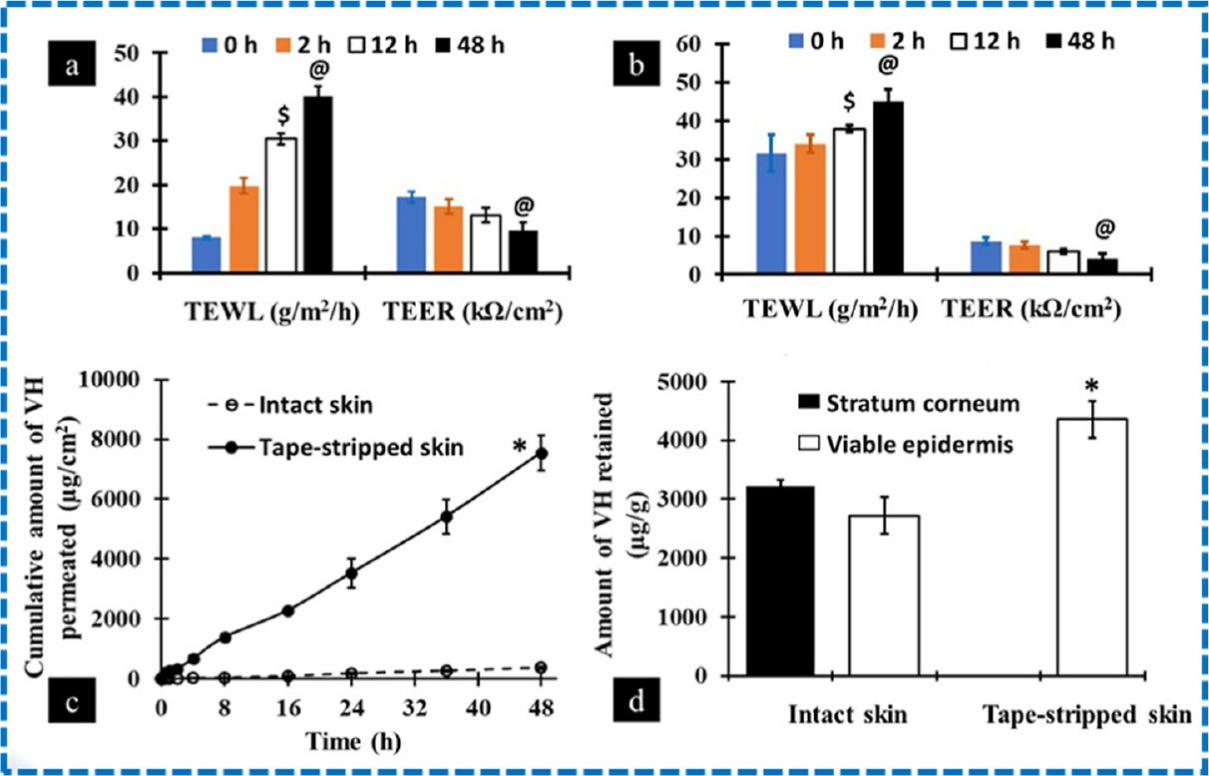
Measurement of TEWL and TEER in intact (a) and tape-stripped skin (b). Ex vivo skin permeation profile (c) and retention (d) of vancomycin across and within the intact and tape-stripped skin. Values represent mean ± SD (*n* = 4). “$” denotes that the TEWL value is significantly different at p < 0.05 compared to 0 and 2 h. “@” depicts that, at 48 h, the TEWL and TEER results differ significantly compared to all other groups at *p* < 0.05. “*” represents that the value is significantly different at *p* < 0.05 compared to the other groups.

Ex vivo skin permeation studies for the VH-loaded CAGE-P gel formulation in the intact and tape-stripped skin are depicted in Figure 7c. The application of CAGE-P gel favored the transport of VH across the intact and tape-stripped skin. The permeation of VH across the intact skin was found to be 369 ± 41 μg/cm^2^, and for the tape-stripped skin, the transport of VH was significantly enhanced to 7543 ± 585 μg/cm^2^ (Table 5). For tape-stripped skin, the flux and permeability coefficients were enhanced by 20.02-fold and 19-fold, respectively, compared to intact skin. Notably, the lag time was calculated as zero for the VH + CAGE-P gel formulation in tape-stripped skin. The amount of VH retained in the SC and viable skin for the intact skin was 3212 ± 108 and 2720 ± 313 μg/g, respectively. After tape-stripping, VH retained in the viable skin was increased by 1.61-fold in comparison to the intact skin (Figure 7d).

**Table 5:** Skin Permeation Parameters of VH after Treatment with CAGE-P-Gel Formulation^a^

treatment groups	*T*_lag_ (h)	*J* (μg/cm^2^/h)	*K*_p_ (×10^–3^ cm/h)	*D* (×10^–3^ cm/h)	*Q*_48_ (μg/cm^2^)
intact skin					
CAGE-P gel	1.01 ± 0.2	7.6 ± 0.8	0.8 ± 0.08	0.38 ± 0.009	369 ± 41
tape-stripped skin					
CAGE-P gel	0	152.2 ± 13.6^a^	15.2 ± 1.6	0	7453 ± 585^b^

a *T*_lag_, lag time; *J*, Flux; *K*_p_, permeability coefficient; *D*, diffusivity; *Q*_48_, the cumulative amount of VH permeated after 48 h per unit area. The thickness of the intact and tape-stripped skin was measured to be 0.32 ± 0.038 mm and 0.17 ± 0.04 mm, respectively. Data represents mean ± SD (*n* = 4). “a” and “b” represent that the flux values, and cumulative amount permeated are significantly different at (*p* < 0.05) for tape-stripped skin compared to the intact skin treatment group. CO—choline/oleic acid; CP—choline/palmitic acid; CAGE-IL—choline geranate ionic liquids; CAGE-P gel—choline geranate Pluronic ionogel.

### 3.9 Mechanistic Studies to Determine the Action of Chemical Enhancers

The TEWL and TEER values before and after the treatment with PBS, CO, CP, CAGE, and CAGE-P gel formulations at two different time points (2 and 12 h) are shown in Tables 6 and 7 for comparison. The values are shown for both intact and tape-tripped skin. The TEWL value for intact porcine skin was less than 10 g/m^2^/h. For the tape-stripped skin, the TEWL value was more than 10 g/m^2^/h. The intact skin treated with the control formulation, PBS, for 12 h, did not show a significant increase in the TEWL values when compared with the 2 h treatment. This observation was the same in the case of tape-stripped skin. However, the application of different enhancers to the skin increased the TEWL values significantly (*p* < 0.05) from 2 to 12 h. Both intact and tape-stripped skin samples treated with CAGE or CAGE-P gel showed the highest TEWL values after 12 h of co-treatment.

**Table 6:** Effect of Permeation Enhancers on TEWL of Excised Porcine Skin^a^

treatment groups	treatment time		
intact skin	0 h	2 h	12 h
CO	7.98 ± 0.62	17.25 ± 2.18	23.48 ± 3.16
CP	7.45 ± 0.74	15.25 ± 1.32	19.33 ± 1.08
CAGE-IL	8.33 ± 1.08	21.10 ± 2.10	26.93 ± 1.41
CAGE-P gel	7.80 ± 0.52	23.40 ± 2.19^a^	27.80 ± 2.06^b^
tape-stripped skin			
CO	22.10 ± 1.48	25.70 ± 1.91	29.98 ± 1.27
CP	22.90 ± 1.57	26.40 ± 1.48	30.68 ± 1.06
CAGE-IL	27.20 ± 1.00	31.70 ± 1.77	36.73 ± 2.03
CAGE-P gel	28.30 ± 0.65	33.30 ± 2.63^a^	38.83 ± 2.25^b^

a CO—choline/oleic acid; CP—choline/palmitic acid; CAGE-IL—choline geranate ionic liquids; CAGE-P gel—choline geranate Pluronic ionogel. Values represent mean ± SD (*n* = 4). For control, the skin was spiked with neat PBS, without any enhancers, for which the TEWL value was in the range of 12–15 g/m^2^/h and 20–25 g/m^2^/h for intact skin and tape-stripped skin, respectively. “a” and “b” indicate that, after 2 and 12 h, respectively, in comparison to all other groups in intact and tape-stripped skin, except CAGE-IL, the values are significantly different at *p* < 0.05.

**Table 7:** Effect of Permeation Enhancers on TEER of Excised Porcine Skin^a^

treatment groups	treatment time		
intact skin	0 h	2 h	12 h
CO	15.45 ± 0.60	13.20 ± 0.70	8.93 ± 0.51
CP	15.05 ± 1.30	13.23 ± 1.03	9.85 ± 0.53
CAGE-IL	15.70 ± 0.70	13.40 ± 0.91	9.25 ± 1.20
CAGE-P gel	14.78 ± 1.23	12.55 ± 1.16	7.95 ± 0.70^a^
Tape-stripped skin			
CO	8.45 ± 0.68	7.85 ± 0.99	6.93 ± 0.85
CP	8.63 ± 0.48	7.93 ± 0.15	6.88 ± 0.61
CAGE-IL	7.85 ± 0.60	7.08 ± 0.82	6.93 ± 0.88
CAGE-P gel	9.28 ± 0.67	8.25 ± 0.48	6.40 ± 0.43^a^

a CO—choline/oleic acid; CP—choline/palmitic acid; CAGE-IL—choline geranate ionic liquids; CAGE-P gel—choline geranate Pluronic ionogel. Values represent mean ± SD (*n* = 4). For control, the skin was spiked with neat PBS, without any enhancers, for which the TEER value was in the range of ≥15 kΩ/cm^2^ and ≤10 kΩ/cm^2^ for intact skin and tape-stripped skin, respectively. “a” indicates that, at 12 h, the values differ significantly from all other groups in both intact and tape-stripped skin, with a *p* < 0.05.

The TEER values for untreated skin samples were more than 15 kΩ/cm^2^. For intact and tape-stripped skin, the TEER values decreased significantly (*p* < 0.05) after co-treatment for 2 h with CAGE-IL. At the end of 12 h, the TEER for CAGE-P gel formulation was decreased by 0.53-fold and 0.42-fold in both intact and tape-stripped skin in comparison to untreated skin samples (Table 7).

Figure 8a–p shows the SEM images captured for the neat skin samples treated with the control formulation and with enhancers for 2 and 12 h. The untreated skin surface for intact skin was relatively smooth compared to tape-stripped skin. The skin samples treated with PBS as a control did not change surface morphology to an extent. Nevertheless, the skin’s surface was changed in various ways for intact and tape-stripped skin after treatment with permeation enhancers. Figure 8g,h,o,p shows that after 12 h of incubation, the skin surface was more cracked and porous as a result of the CAGE and CAGE-P gel treatment.

**Figure 8:**
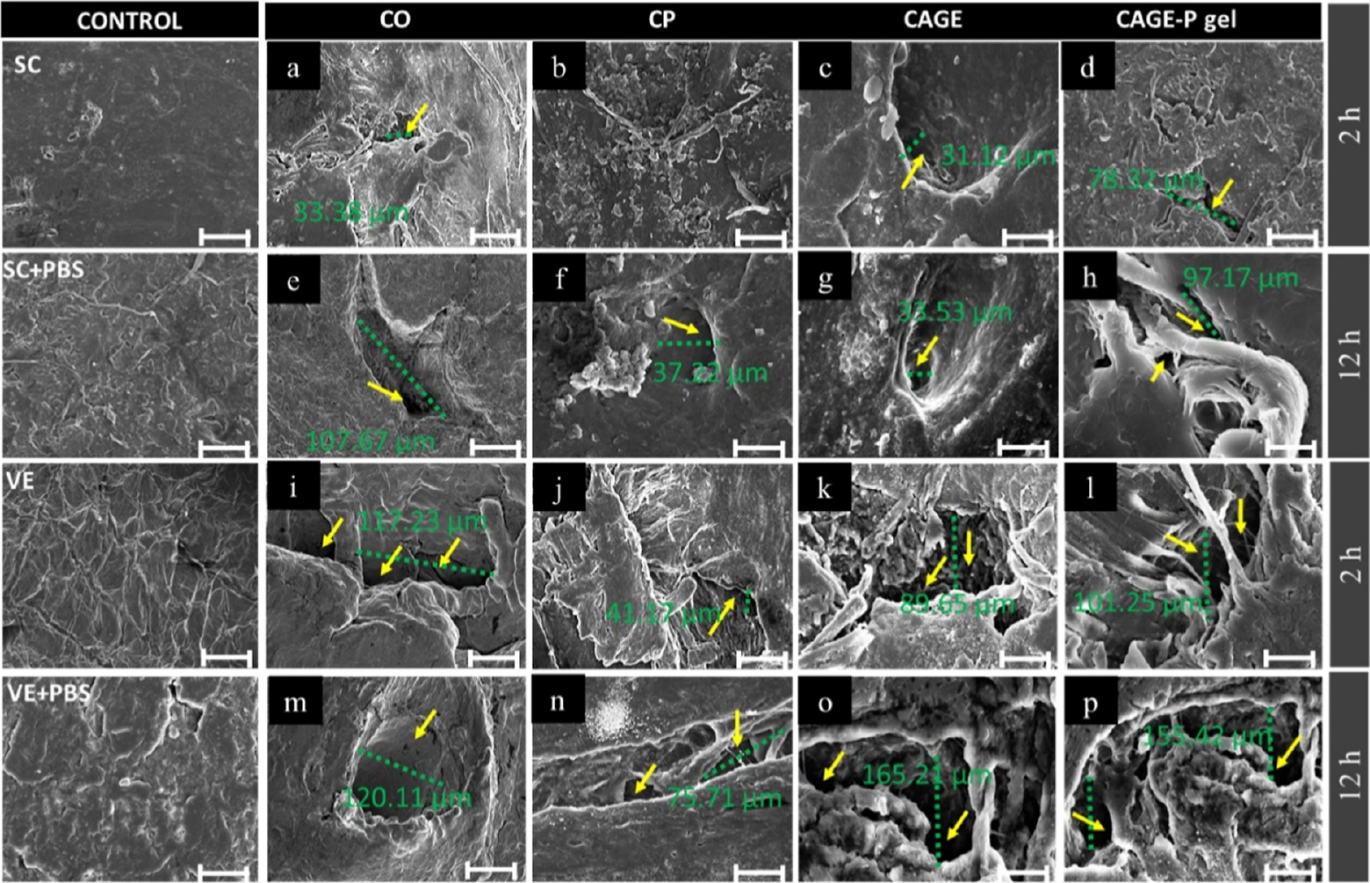
SEM images showing surface morphology for intact SC and viable epidermis (VE) treated with different formulations at different time points. The left panel shows the neat SC and VE, with their respective control groups, treated with phosphate-buffered saline (PBS, pH 7.4) at 0 h. SEM images were captured for SC (a–d) and VE (i–l) treated at 2 h, and SC (e–h) and VE (m–p) treated at 12 h. The yellow arrow indicates the presence of cracks or wide pores on the surface, which were more prominent for the CAGE and CAGE-P gel-treated groups. The green line indicates the representative measurement of pore size in micrometers. The scale bar indicates 50 μm.

The data for the thermal examination of skin samples treated with PBS, CO, CP, CAGE, and CAGE-P gel formulations for 2 and 12 h are shown in Table 8 and Figure 9a,b. The incubation of SC and VE with PBS did not significantly (*p* < 0.05) decrease the transition temperature or enthalpy when compared to untreated SC and VE samples. Untreated SC samples showed a transition temperature of 68.32 ± 1.56 °C. In general, the transition temperature of skin samples was decreased after 12 h of incubation with CO, CP, and CAGE-IL formulations. At 12 h, the CAGE-P gel formulation showed a notable (*p* < 0.05) drop in transition temperature. The same pattern was observed for the tape-stripped skin samples treated with different enhancers at 2 and 12 h. In general, Δ*H* decreased after treatment with CO, CP, CAGE, and CAGE-P gel formulations. However, the decrease was significant (*p* < 0.05) after incubation with CAGE and CAGE-P gel formulations at 12 h compared to 2 h and untreated skin for both intact and tape-stripped skin.

**Table 8:** Thermal Analysis of SC and Viable Skin before and after Treatment with Different Permeation Enhancers^a^

treatment groups	thermal parameters and treatment time			
	transition temperature (°C)		enthalpy change (Δ*H*, J/g)	
intact skin	2 h	12 h	2 h	12 h
CO	70.70 ± 2.81	63.04 ± 4.90	–598.12 ± 25.14	–488.60 ± 45.83
CP	70.86 ± 2.20	64.01 ± 3.50	–624.23 ± 12.81	–568.67 ± 50.93
CAGE-IL	62.00 ± 4.00	61.69 ± 5.28	–478.97 ± 86.19	–467.75 ± 19.60
CAGE-P gel	66.47 ± 5.56	60.82 ± 3.26	–493.07 ± 26.05^a^	–441.87 ± 36.02^b^
tape-stripped skin				
CO	62.75 ± 4.66	60.54 ± 4.22	–528.73 ± 59.33	–393.83 ± 60.59
CP	62.05 ± 3.17	60.61 ± 3.50	–540.07 ± 34.59	–363.90 ± 47.84
CAGE-IL	60.11 ± 3.22	66.41 ± 5.14	–430.80 ± 31.94	–299.72 ± 56.58
CAGE-P gel	64.21 ± 5.54	64.41 ± 4.01	–416.50 ± 47.92^a^	–281.19 ± 31.88^c^

a CO—choline/oleic acid; CP—choline/palmitic acid; CAGE-IL—choline geranate ionic liquids; CAGE-P gel—choline geranate Pluronic ionogel. Values represent mean ± SD (*n* = 3). “a” indicates that, except for CAGE-IL after 2 h, the value is significantly different at *p* < 0.05 relative to all the other groups. “b” and “c” indicate that the value is significantly different at *p* < 0.05 compared to CP and CO treated group, respectively after 12 h. The transition temperature for SC and viable epidermis for intact and tape-stripped skin was found to be 68.32 ± 1.56 °C and 70.8 ± 0.81 °C, respectively. The enthalpy change for SC and viable epidermis for intact and tape-stripped skin was found to be −654.12 ± 15.32 J/g and −649.5 ± 24.81 J/g, respectively. For control, both the SC and VE were spiked with neat PBS without any enhancers. For SC, the transition temperature and enthalpy change were found to be 67.02 ± 3.01 °C and −647.23 ± 12.34 J/g, respectively. For VE, the transition temperature and enthalpy change were found to be 68.75 ± 4.13 °C and −641.10 ± 22.13 J/g, respectively.

**Figure 9:**
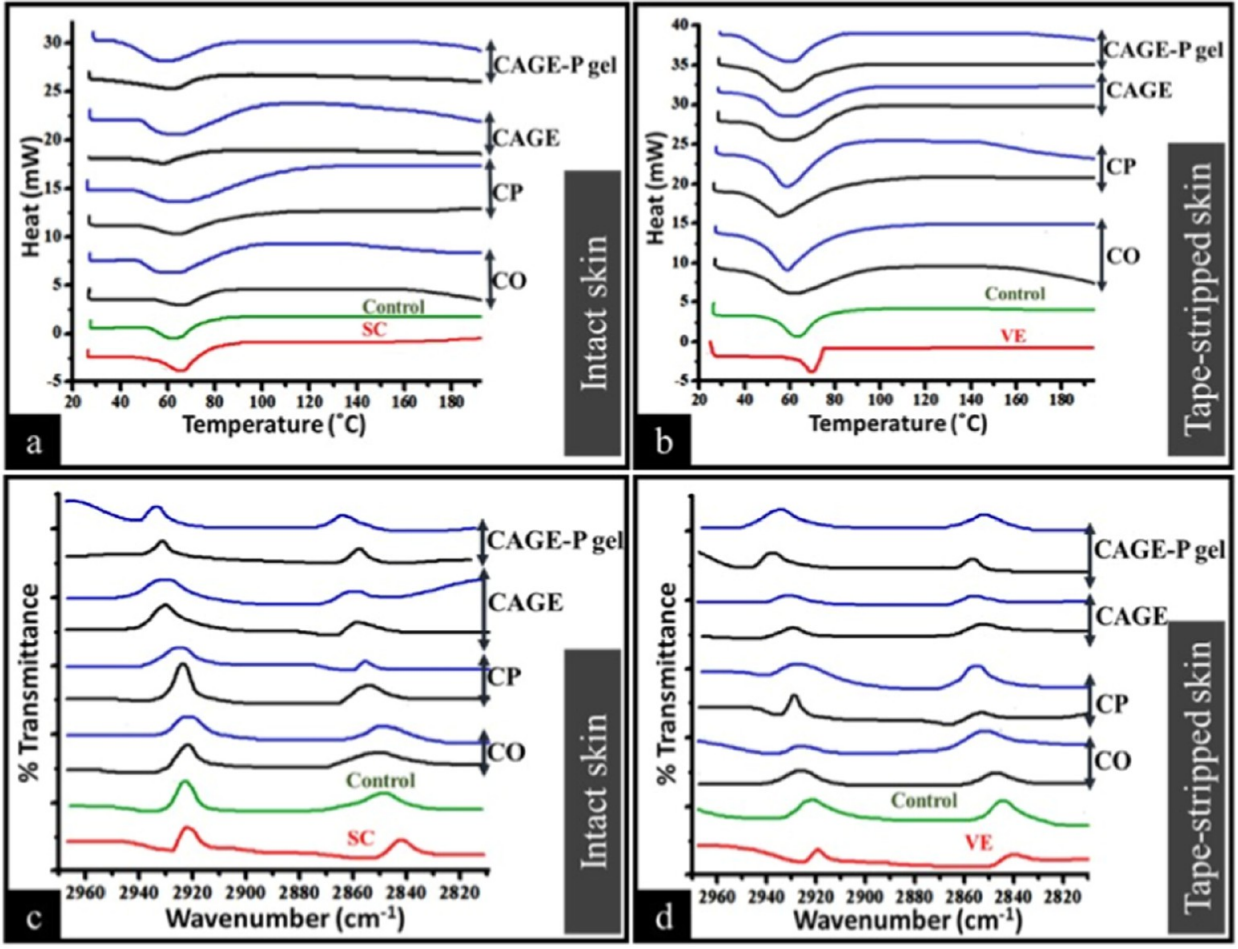
Representative DSC thermograms (a,b) and FT-IR spectra (c,d) of intact (SC), and tape-stripped skin (VE), respectively. Both SC and VE were treated with different formulations for 2 h (black line) and 12 h (blue line), respectively. For control, in both cases, SC and VE were treated with PBS, pH 7.4.

The FT-IR spectra of SC and VE are shown in Figure 9c,d, respectively, before and after treatment with enhancers at various time intervals. Asymmetric C–H stretching (2920 cm^–1^) and symmetric stretching (2850 cm^–1^) were detected in the lipids. After the enhancers were applied, a change to higher wavenumbers was seen. Peak positions were not significantly altered when SC or VE were treated with PBS as compared to untreated SC or VE. The peak height at 2920 cm^–1^ and 2850 cm^–1^ was lowered in the SC or VE samples incubated with CAGE and CAGE-P gel formulations after 12 h, suggesting lipid extraction.

### 3.10 In Vivo Skin Irritation Analysis

SD rats were anaesthetized with 5% isoflurane and 21% oxygen and assessed for LDF and TEWL on their intact and tape-stripped skin. LDF monitors blood flow to continuously assess skin irritation caused by penetration enhancers. The normal blood flow rate in rats was 64.75 ± 5.09 mL/min (Figure 10a). The use of VH + CAGE and VH + CAGE-P gel raised blood flow rates to 78.42 ± 4.96 mL/min and 80.3 ± 5.1 mL/min, respectively. Compared to intact skin, tape-stripped skin with VH + CAGE or VH + CAGE-P gel formulations dramatically enhanced blood flow rate (*p* < 0.05) (Figure 10b). Figure 10a,b shows the TEWL values of the rat skin after 2 h of the application of VH in CAGE and CAGE-P gel formulations. The application of VH in CAGE and CAGE-P gel formulations for 2 h increased TEWL by 1.31-fold and 1.40-fold in tape-stripped skin compared with the untreated tape-stripped skin. The calculation of the mean skin blood flow and skin integrity assessed by LDF and TEWL, respectively, was based on mean ± SD, *n* = 4, measurements.

**Figure 10:**
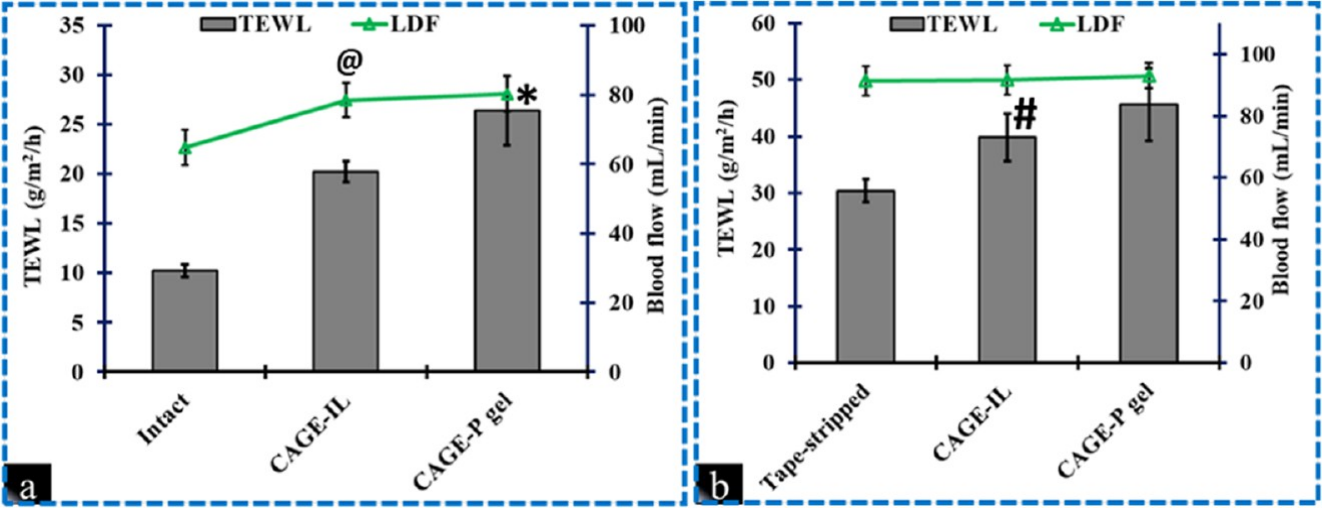
TEWL and LDF values for intact (a) and tape-stripped (b) skin treated with VH-loaded in CAGE and CAGE-P gel formulations after 2 h of application. “@” and “*” denote that the value for CAGE-IL and CAGE-P gel is significantly different at p < 0.05 compared to untreated intact skin. “#” denotes that the value for CAGE-IL is significantly different at *p* < 0.05 compared to untreated tape-stripped skin. Values are represented as mean ± SD; n = 4. CAGE-IL—choline geranate ionic liquid; CAGE-P gel—choline geranate Pluronic ionogel.

Figure 11a–c shows the digital images captured for the healthy and excised dorsal skin of SD rats after tape-stripping. Figure 11d–i show the H&E-stained microscopic images of the cryosectioned intact and tape-stripped rat skin before and after the application of different VH formulations. The control group signifies healthy rat skin, distinguished by an intact SC and epidermal layer. The tape-stripped group showed the removal of all SC layers, with an exposed epidermal layer. The groups administered with the VH + CAGE-IL formulation displayed no indications of epidermal thickening or keratinocyte hyperproliferation, showing equivalence to the untreated or intact skin. No significant damage was induced upon the application of VH-loaded CAGE or CAGE-P gel on the tape-stripped skin. Interestingly, after 14 days, LDF and TEWL were measured, which showed a decrease in the skin blood flow and water loss (Figure 11j). Encouraged by these results, the excised skin samples were subjected to H&E staining that showed the reversible, partial, and loose appearance of SC for the tape-stripped skin applied with VH + CAGE-P gel formulation (Figure 11k).

**Figure 11:**
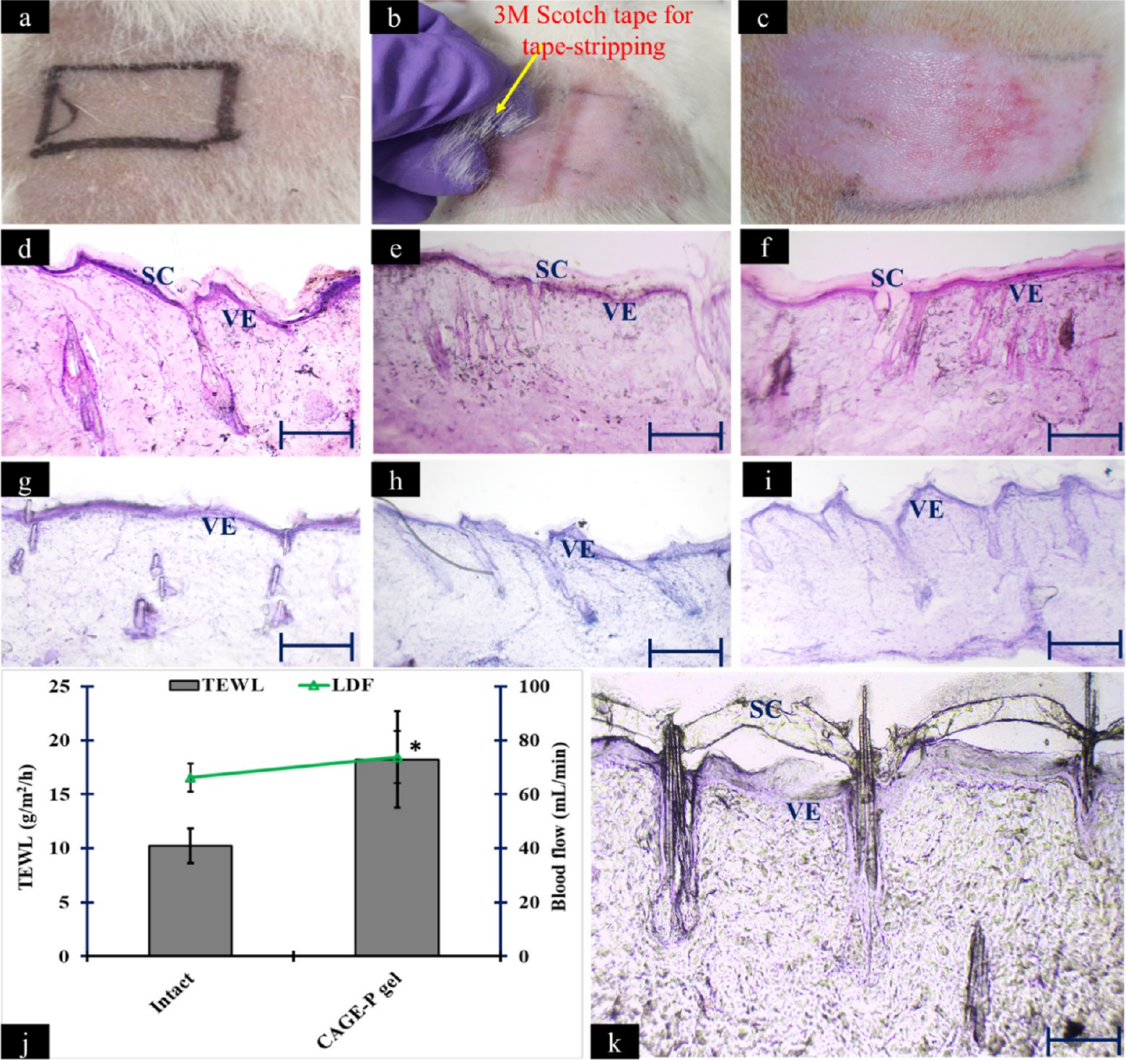
Representative digital images were captured for the shaved dorsal skin without furrows of SD rats (a), tape-stripping with 3M scotch tape for the removal of the SC (b), and morphological appearance of skin after tape-stripping (c). H&E-stained images were captured for the intact skin (d), and intact skin, applied with VH + CAGE (e) and VH + CAGE-P gel formulations (f). H&E-stained images were captured for the tape-stripped skin (g), and tape-stripped skin, applied with VH + CAGE (h) and VH + CAGE-P gel formulations (i). Measurement of TEWL and LDF after 14 days for the tape-stripped skin that was applied with VH + CAGE-P gel formulation. “*” indicates that the value for VH + CAGE-P gel is significantly different at *p* < 0.05 compared to the intact skin (j). H&E-stained images were captured for the tape-stripped skin applied with VH + CAGE-P gel formulation after 14 days (k). The scale bar indicates 100 μm.

## 4 Discussion

Chemical enhancers in transdermal drug delivery systems have been employed for decades to augment drug permeation. To this end, a mixture of conventional fatty acids (oleic acid, OA and palmitic acid, PA) and choline geranate-based ILs (CAGE-IL) was investigated in our current work. From a mechanistic perspective, these enhancers, OA and PA, can augment penetration by enhancing the fluidity of the SC intercellular lipids or modifying the structure of skin proteins.^37^ Conversely, ILs have been shown as a feasible enhancer to solubilize hydrophilic compounds to enhance skin permeation^38,39^ modifying the skin barrier function, SC. Based on these selected and optimized enhancers, this study also offered a comprehension of the mechanics underlying the dynamic actions in altering the skin integrity in intact and tape-stripped skin.

OA and PA alter the organized lipid bilayer interspersed among the corneocytes of the SC. These integrate into lipids, produce phase transitions, and enhance fluidity.^40–43^ CB and GA are cationic and anionic entities with a molecular weight of 165.19 and 168.23 g/mol, respectively. A salt metathesis reaction takes place between the CB and GA, involving the exchange of bonds and the formation of a product with similar or identical affiliations that result in the generation of a CAGE. CB and OA or PA were mixed in a molar ratio of 2:1, which resulted in the formation of choline/OA (CO) and choline/PA (CP) mixtures. This molar ratio was selected to enhance the bulkiness of the cationic groups, which can have a better interaction with the negative charge of the skin. However, the zeta potential showed a negative surface charge for both formulations. In the study reported by Tahara et al., among various selected fatty acids, OA in combination with CB showed an enhanced penetration (0.2 μg/cm^2^) for water-soluble antigen peptide across an intact whole pig skin.^44^ Our studies also showed the application of CO in the enhanced transport of water-soluble VH across the intact porcine skin. This can be attributed to the pKa/pH and charge correlation. CB is reported to have a pKa value of 13.9, which is more than the pH of the SC surface (4.5–5.5), which will facilitate cutaneous penetration through the intercellular route to the SC surface, however, their diffusion will be limited at the SC interface. Based on the charge of the CO (−3.01 ± 0.8 mV), the electrostatic repulsion was less, and therefore, the amount permeated in the SC was found to be (133 ± 14 μg/cm^2^). In a different set of experiments, where CP was applied, the permeation was significantly (*p* < 0.05) less (95 ± 6 μg/cm^2^) in comparison to CO, which may be due to the highest electrostatic repulsion (−28.39 ± 3.67 mV) as evident from the zeta potential studies. CAGE with a minimal zeta value (−0.067 ± 0.0023 mV) showed enhanced VH permeation (278 ± 49 μg/cm^2^). Taken together, more negatively charged formulations showed an electrostatic repulsion with the negatively charged lipids present in the SC.^45,46^ Gustafson et al. studied charge–charge interaction for the positively charged VH with that of oligo (poly­(ethylene glycol) fumarate (OPF)) and sodium methacrylate (SMA) UV-crosslinked polymeric films.^47^ The positively charged SMA, with an increase in the ratio upon interaction with positively charged VH, showed delayed and extended release for 4 days. This study was limited to the investigation of the properties of the OPF/SMA hydrogel film alone.

The penetration of VH was examined in tape-stripped skin. The SC serves as the principal barrier for the transdermal transport of bioactive compounds.^48^ Removing SC would increase the VH penetration, according to the hypothesis. The removal of SC was accomplished by the physical process of tape-stripping. Following a combination of VH + CO or CP on intact skin, the penetration coefficients were 0.00027 and 0.00018 cm/h, respectively. Due to improved VH partitioning in hydrated skin and the lack of the lipidic SC barrier, the permeability coefficient of VH is much higher in tape-stripped skin compared to intact skin.^49^ In line with our earlier research on VH permeation, it was intriguing to observe that the penetration of VH in intact skin was noticeably greater (*p* < 0.05) following co-treatment with neat CO and CP compared to neat applications of OA and PA. Importantly, VH penetration across tape-stripped skin was significantly improved by CO as well. Nevertheless, when tested on tape-stripped skin, CP demonstrated much reduced (p < 0.05) penetration compared to PA application. CAGE was the most effective in increasing penetration of VH, compared to any other group.

The optimized CAGE formulation was integrated within the polymeric matrix of Pluronic F-127 to prepare an organogel. Organogel formulations have been documented well for the ease of spreading and enhancement of the molecules due to their shear-thinning characteristics and more organic or organogelator content.^50,51^ In our studies, organogel has been colloquially termed an ionogel formulation due to the incorporation of CAGE (a mixture of cation and anion).^52^ Ionogels are fascinating materials composed of crosslinked polymeric networks swollen with ILs.^53–55^ The VH + CAGE-P ionogel was clear and stable without phase separation. The contact angle of the prepared gel formulation was less, and the viscosity of the fabricated VH-loaded ionogel made of Pluronic F-127 and PEG-400 showed a desirable viscosity of 3050 mPa·s at a shear rate and temperature of 500 s^–1^ and 25 °C, respectively. This showed that the gel formulation was more consistent, making it easier to apply and spread over the skin.^56^

The 5% concentration of VH in the CAGE-P ionogel yielded the highest flux and cumulative permeation across both intact and tape-stripped skin. Furthermore, the amount of VH retained in the SC and viable skin was superior when VH was administered by using a CAGE-P ionogel. The current work demonstrated that the highest flux and cumulative permeation of VH across the skin was attained with CAGE-P ionogel, surpassing previous investigations with alternative thermosensitive gelling systems. In the study reported by Mahmoudian and Ganji, VH was encapsulated in the polymeric HPMC microparticle by the spray-drying process.^57^ A composite VH release platform was created by embedding VH-HPMCS in chitosan/glycerophosphate (CH/GP). VH release was sustained for 160 days, with a percentage cumulative release of 85% from the HPMCs/CH/GP thermosensitive gel matrix. This delivery system was designed to locally treat osteomyelitis with a long-term release profile to reduce the injection frequency. Another study showed the preparation of thermosensitive gel using poly­(ethylene glycol) diacrylate and poly­(*N*-isopropylacrylamide) by polymerization techniques.^58^ In vitro release studies showed a 36% initial burst of VH within 24 h, followed by 84% in the next 504 h (21 days). The formulation was studied in vivo for the treatment of ocular surgery and showed a release profile for 21 days. Notably, VH increased penetration in an ionogel containing CAGE, Pluronic F-127, and poly­(ethylene glycol) (PEG) compared to pure CAGE. Both with and without Pluronic F-127 gel, CAGE acts on VH and skin membranes. The incorporation of permeation enhancers into the skin membrane is more effective when diluted in an appropriate vehicle than in an undiluted, neat form.^59,60^

Furthermore, the dynamic effect of CO, CP, CAGE, and CAGE-P ionogel on the skin lipids was also investigated with TEWL, TEER, SEM, DSC, and FT-IR studies. Some references used chemical enhancers to structurally modify skin lipids to increase drug absorption.^61^ From the DSC and FT-IR results, all of the formulations showed significant influence on the variations of the wavenumber and transition temperature. CAGE or CAGE-P ionogel treatment of skin resulted in the highest enthalpy shift and percent change compared with CO and CP. These shifts usually suggest a more disordered organization of SC intercellular lipids. A possible explanation for the variations in the skin’s transition temperature is the disruption and subsequent fluidization of lipid bilayers.^62^ Similarly, the degree of change in the TEWL and TEER values was significantly different for the skin samples treated for 12 h with CAGE and CAGE-P ionogel for intact or tape-stripped skin samples. In addition, SEM studies showed the enlargement of pores created on the skin surface with an increase in the incubation time on co-treatment with different enhancers. The formation of such pores or microchannels is the probable pathway for the increased permeation of the drug molecules.^63–65^ Herein, the skin lipids and enhancers’ intermolecular forces were predicted using the solubility parameter. Using the Hansen solubility parameter, the enhancers’ solubility parameters were ascertained.^66^ This parameter is a composite outcome of three rigorous intermolecular forces, namely, dispersion forces, polar forces, and hydrogen bonds. A comparable solubility parameter indicates a favorable miscibility to a certain extent.^67,68^ The solubility parameter (δ) calculated for CB and GA was 4.626 (cal/cm^3^)^1/2^ and 7.013 (cal/cm^3^)^1/2^, respectively, which was nearer to the solubility parameter of VH (δ = 6.094 (cal/cm^3^)^1/2^). In comparison to CO and CP, CAGE exhibited a greater solubility parameter (δ = 11.639 (cal/cm^3^)^1/2^), nearest to the solubility parameter of skin (δ = 10 (cal/cm^3^)^1/2^), signifying the prominent intermolecular force.^60^ The higher miscibility suggested a higher lipid disorder ability, which finally caused the highest enhancement ratio (ER) in the intact or tape-stripped skin (Figure S8).

The changes in the LDF and TEWL data were significantly greater for the tape-stripped skin compared with intact skin after the application of different formulations on the dorsal surface of the SD rats. Increased redness of the skin was observed after tape-stripping, which increased microcirculation with the enhanced transportation of VH with enhancers. The application of the CAGE solution was found to induce a noticeable increase in the skin microcirculation. The application of CAGE-P gel did not significantly increase microcirculation and TEWL, which can be attributed to the presence of PEG in a gel-based carrier that has an emollient activity when applied to the skin.^69^ Interestingly, after 14 days of observation, the skin condition was reversed and the measured LDF and TEWL were decreased. The pairing of ions, which diminishes net conductivity, leads to charge shielding and subsequently lessens skin irritation potential; therefore, ILs with lower conductivities are favored to mitigate skin irritation.^33^ To this end, our studies showed the conductivities of CAGE and VH-loaded in CAGE-P ionogel to be 2.55 ± 0.209 and 6.69 ± 0.221 mS/cm, respectively.

Based on the above findings, we concluded that the selection of the optimized permeation enhancer, CAGE, was a key step for the enhancement of a model hydrophilic and high-molecular-weight drug, VH. Nevertheless, the combination of fatty acids with ILs can also be explored to enhance the permeation to a certain extent. The findings from the mechanistic studies can be key indicators of enhancers to characterize the interaction between enhancers and skin lipids. We envision that further in vivo studies could be a potential strategy for the clinical establishment of VH-based ionogel that requires high-dose administration. Taken together, when compared to alternative minimally invasive or invasive methods, the topical delivery of VH in CAGE-P ionogel is the most straightforward, non-invasive, and simple method.

## 5 Conclusions

Choline-based ILs in ionogels transported the hydrophilic macromolecule VH through the skin in this investigation. The fabricated choline-based ILs, CO, CP, and CAGE, showed homogeneous preparations with pH similar to physiological skin conditions. We used a mixture of unsaturated OA (C18) and saturated PA (C16) as enhancers with CB. Choline geranate was made from GA and CB. In vitro experiments showed that neat VH dissolved in phosphate-buffered saline at pH 7.4 and CAGE diffused better due to lower viscosity, which expedited drug release. In contrast, the CAGE-P ionogel released VH more slowly. Neat VH did not permeate excised porcine skin in an ex vivo skin penetration study. However, CAGE, followed by choline/OA, increased permeability in both intact and tape-stripped skin. Moreover, adding CAGE to the Pluronic F-127 ionogel improved VH skin permeability. Enhanced transdermal transport of VH can be achieved by many mechanisms. These include VH solubilization by CAGE and CAGE-P ionogel, skin barrier changes, and lipid matrix extraction or interference. After CAGE and CAGE-P ionogel formulations were used, biophysical studies showed skin barrier changes. In vivo skin irritation investigations showed increased TEWL, blood flow, and other skin barrier alterations. Pharmacokinetic studies can translate the VH + CAGE-P ionogel’s effectiveness to clinical trials. Nonetheless, this study deepened our understanding of how choline-based ILs and formulated CAGE-based ionogels work in greatly enhancing the transdermal delivery of VH across intact and tape-stripped skin.

## Supplementary Material



## Notes

Scheme for the preparation of ILs and ILs containing vancomycin in poloxamer gel; digital images for the prepared IL formulations; zeta potential and conductivity measurements for the prepared IL formulations; docking studies of prepared IL formulations with skin protein; digital images and microscopic images captured for the skin separation process and corneocytes distribution, respectively; digital images captured for the separation of SC from viable epidermis; swelling, porosity and SEM images for the ionic gel at different time intervals in phosphate buffered saline, pH 7.4; and computation of ER of VH across the tape-stripped skin with and without enhancers (PDF)
