# Supplementary material for: Influence of Choline-Based Ionogel on Transdermal Delivery of Vancomycin Hydrochloride
Source: Mol Pharm. 2025 May 16;22(6):3331–50. doi: 10.1021/acs.molpharmaceut.5c00255 (PMC12135064; doi:10.1021/acs.molpharmaceut.5c00255)
Supplement: Supplementary file 1 [file mp5c00255_si_001.pdf]

Influence of choline-based ionogel on transdermal delivery of vancomycin hydrochloride

Deepanjan Datta[1,2]*, Sony Priyanka Bandi[2], Venkata Vamsi Krishna Venuganti[2]*

[1]Department of Pharmaceutics, Manipal College of Pharmaceutical Sciences, Manipal Academy of Higher Education, Manipal 576104, Karnataka State, India

[2]Department of Pharmacy, Birla Institute of Technology and Science (BITS) Pilani, Hyderabad Campus, Hyderabad 500078, Telangana State, India

***Corresponding author**

Deepanjan Datta, PhD
Assistant Professor
Department of Pharmaceutics
Manipal College of Pharmaceutical Sciences,
Manipal Academy of Higher Education, Manipal 576104, Karnataka State, India
Email: deepanjan.datta@manipal.edu; deepanjandtt@gmail.com
Ph: +91-9102514454

Venkata Vamsi Krishna Venuganti, PhD
Department of Pharmacy, Birla Institute of Technology and Science (BITS) Pilani,
Hyderabad Campus, Hyderabad 500078, Telangana State, India.
Email: vamsi@hyderabad.bits-pilani.ac.in
Ph: +91-4066303581

Supplementary Figures

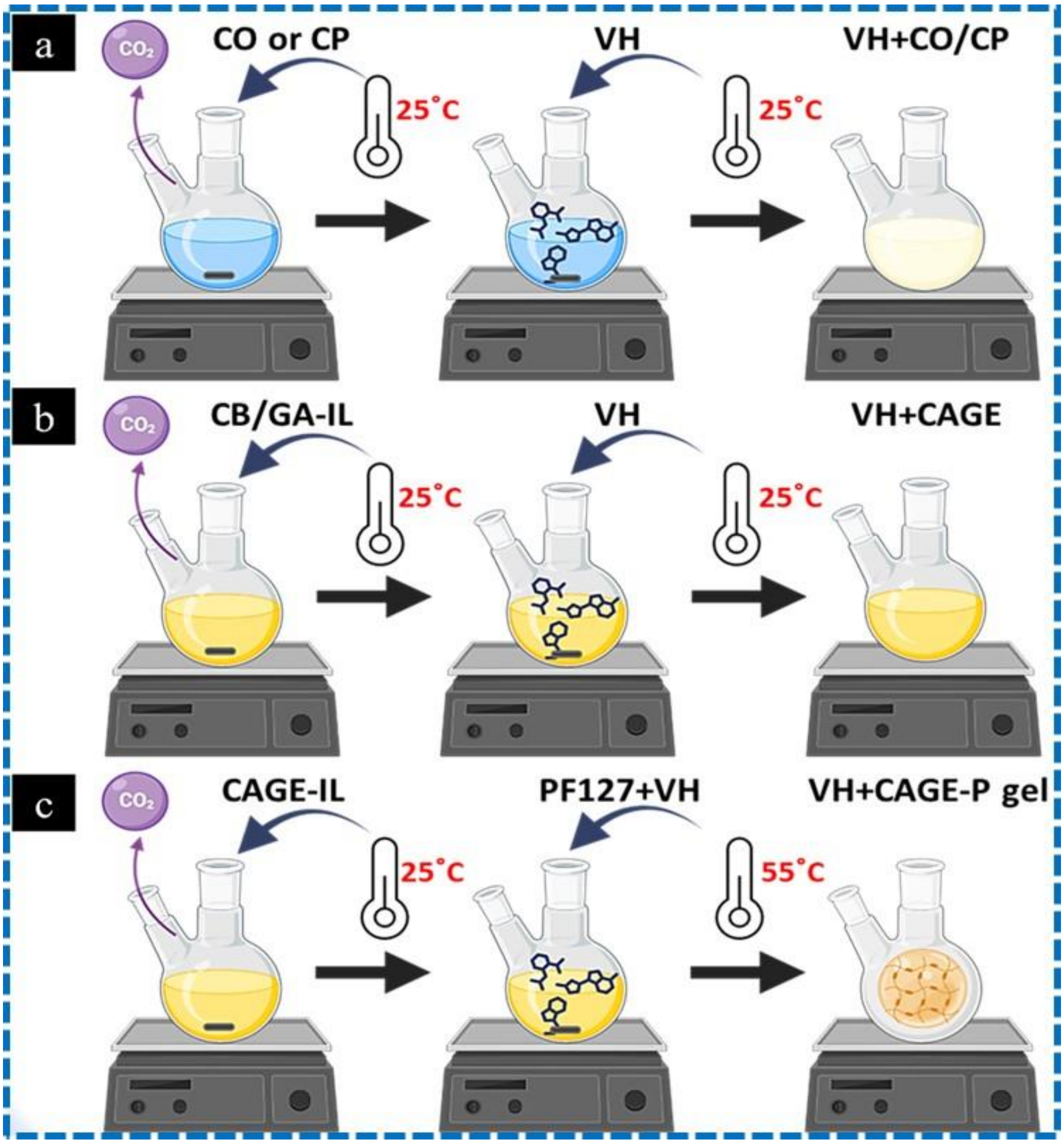

Figure S1. Schematic representation for the preparation of choline/oleic acid (CO) and choline/palmitic acid (CP) (a), choline geranate (CAGE) solution (b) and CAGE-IL Pluronic F-127 ionogel formulation (c) in combination with vancomycin hydrochloride (VH). This image has been created as a creative common using the Biorender software (https://www.biorender.com/).

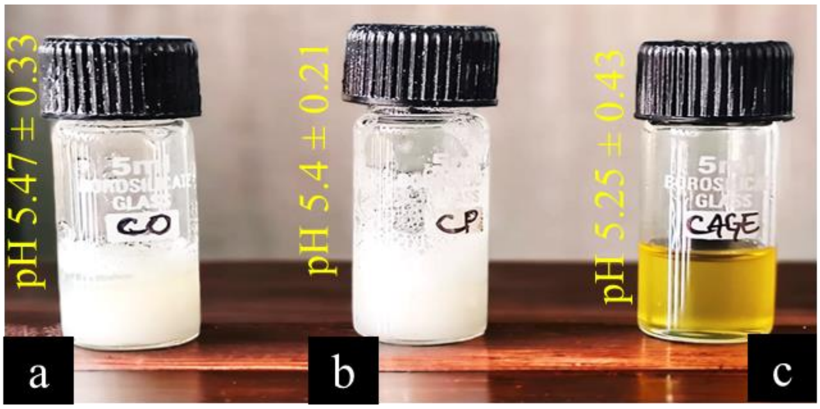

Figure S2. Digital images captured for different VH formulations in choline/oleic acid (CO) (a), choline/palmitic acid (CP) (b), and choline geranate (CAGE) (c) with different pH measurements (mean ± SD, n = 4).

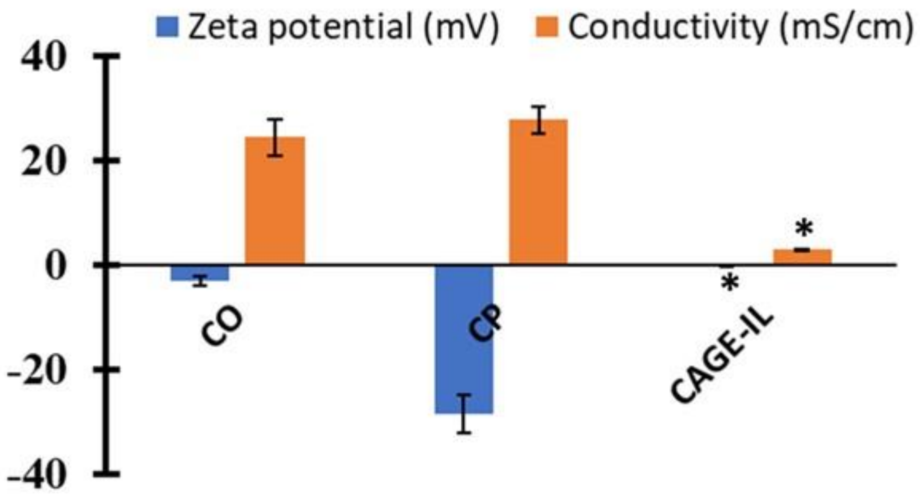

Figure S3. Measurement of zeta potential and conductivity for the mixture of neat enhancers. Values represent mean ± SD (n =3). '*' denotes that the value is significantly different at $p < 0.05$ compared to all the other groups. **CP**−choline/palmitic acid; **CO**−choline/oleic acid; **CAGE-IL**−choline geranate ionic liquid.

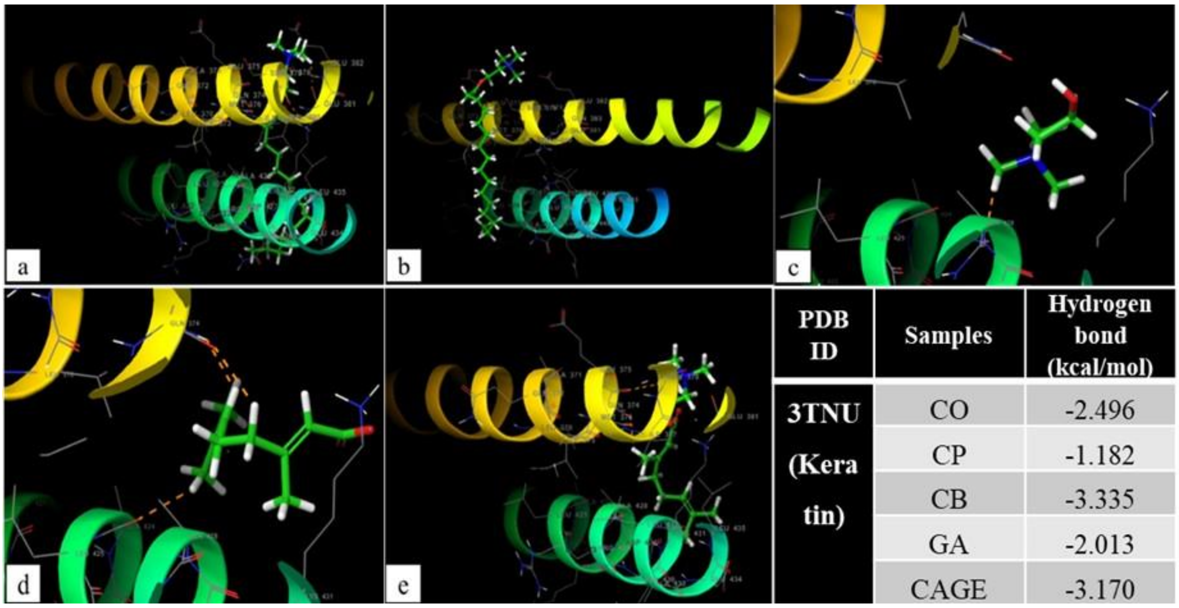

PDB ID	Samples	Hydrogen bond (kcal/mol)
3TNU (Keratin)	CO	-2.496
	CP	-1.182
	CB	-3.335
	GA	-2.013
	CAGE	-3.170

Figure S4. 3D view of interactions of the reported ligand: CO (a), CP (b), CB (c), GA (d) and CAGE (e) with the keratin protein 3TNU. The inset table represents the docking scores (hydrogen bond, kcal/mol) upon the interaction of keratin protein 3TNU with various ligands (CO, CP, CB, GA, and CAGE).

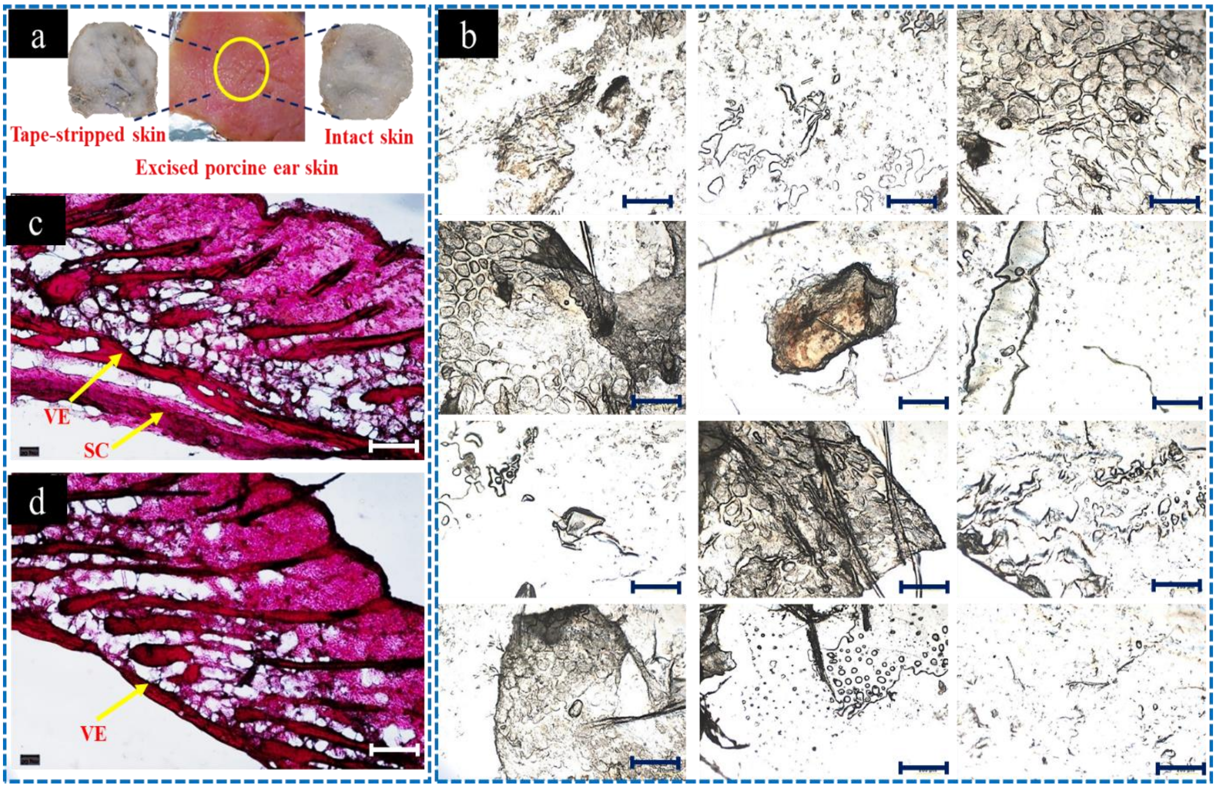

Figure S5. Digital images were captured for excised porcine ear skin with intact and tape-stripped skin (a). Distribution of corneocytes adhering to the 3M Scotch tape upon subsequent

stripping from 1-12 (stripes arranged from left to right) by finger pressure (b). H&E-stained images of cryosections of porcine skin before (c) and after the removal of SC (d). The scale bar represents 200 μm. **SC**−stratum corneum; **VE**−viable epidermis.

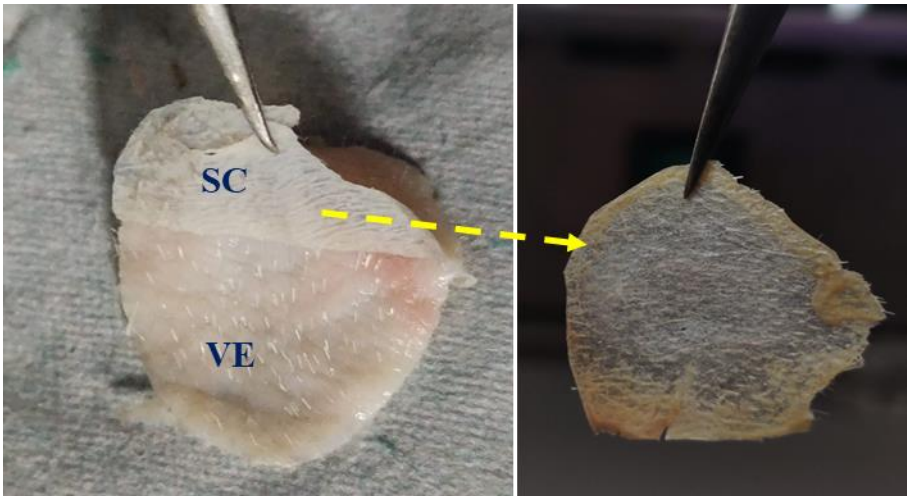

Figure S6. Representative digital images depicting the separation of SC from the VE (viable skin), following pre-treatment of the excised intact skin at 60°C for 90 s in warm water.

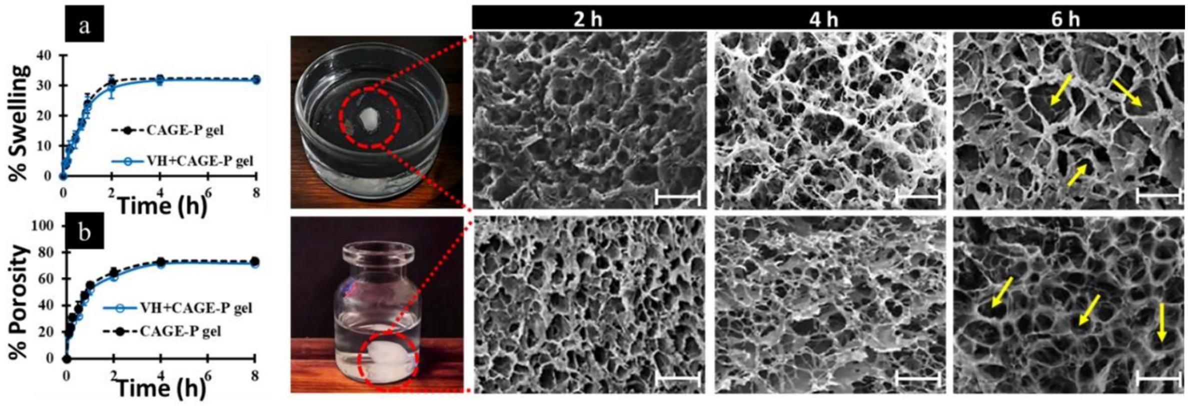

Figure S7. Swelling (a) and porosity (b) behaviour of blank CAGE-P and VH+CAGE-P ionogel performed in PBS, pH 7.4 (37˚C) and IPA, respectively. Data represent mean ± SD (n=4). Digital images of the swollen gel were captured for both swelling (upper panel) and porosity studies (lower panel). Scanning electron microscopic images were captured for VH+CAGE-P ionogel at different time intervals. The yellow arrow indicates the formation of pores after 6 h for the swollen ionogel formulation. Scale bar indicates 100 μm for the images captured at 2 h and 4 h. Scale bar indicates 50 μm for the images captured at 6 h.

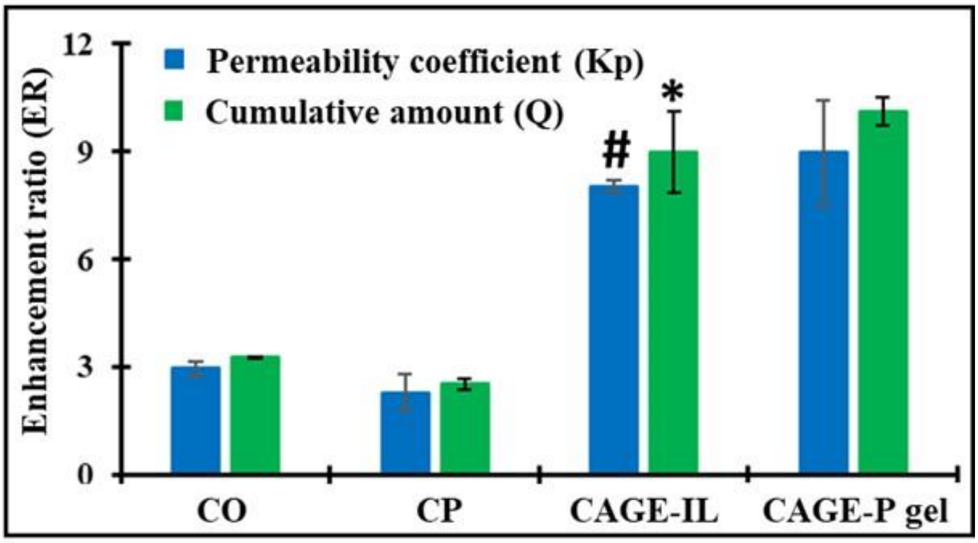

Figure S8. Enhancement ratio (ER) after co-treatment for 48 h was calculated by dividing the permeability coefficient of VH in the presence of CO, CP, CAGE or CAGE-P gel by the permeability coefficient of VH in the absence of enhancers in tape-stripped skin. "*" and "#" represent that the value is significantly different ($p < 0.05$) for tape-stripped skin treated with CAGE for 48 h when compared with the other groups for Kp and Q, respectively. **CO**−choline/oleic acid; **CP**−choline/palmitic acid; **CAGE-IL**−choline geranate ionic liquids; **CAGE-P gel**−choline geranate Pluronic ionogel.
